# Colorectal Polyp Segmentation Based on Deep Learning Methods: A Systematic Review

**DOI:** 10.3390/jimaging11090293

**Published:** 2025-08-27

**Authors:** Xin Liu, Nor Ashidi Mat Isa, Chao Chen, Fajin Lv

**Affiliations:** 1 School of Electrical and Electronic Engineering, Engineering Campus, Universiti Sains Malaysia, Pulau Pinang 14300, Malaysia; liuxin@student.usm.my (X.L.);; 2 School of Automation and Information Engineering, Sichuan University of Science and Engineering, Yibin 644000, China; 3 College of Biomedical Engineering, Chongqing Medical University, Chongqing 400016, China; fajinlv@sohu.com

**Keywords:** polyp segmentation, deep learning, Mamba

## Abstract

Colorectal cancer is one of the three most common cancers worldwide. Early detection and assessment of polyps can significantly reduce the risk of developing colorectal cancer. Physicians can obtain information about polyp regions through polyp segmentation techniques, enabling the provision of targeted treatment plans. This study systematically reviews polyp segmentation methods. We investigated 146 papers published between 2018 and 2024 and conducted an in-depth analysis of the methodologies employed. Based on the selected literature, we systematically organized this review. First, we analyzed the development and evolution of the polyp segmentation field. Second, we provided a comprehensive overview of deep learning-based polyp image segmentation methods and the Mamba method, as well as video polyp segmentation methods categorized by network architecture, addressing the challenges faced in polyp segmentation. Subsequently, we evaluated the performance of 44 models, including segmentation performance metrics and real-time analysis capabilities. Additionally, we introduced commonly used datasets for polyp images and videos, along with metrics for assessing segmentation models. Finally, we discussed existing issues and potential future trends in this area.

## 1. Introduction

Colorectal cancer (CRC) is one of the most common cancers [1]. Most CRC cases develop from small polyps protruding from the surface of the colon and rectum [2]. Colonoscopy can help doctors detect and remove polyps before they turn into cancer. The low contrast of colonoscopy images and the diversity in the size, shape, texture, color, and orientation of polyps pose challenges for precise segmentation [3]. Accurate polyp segmentation is crucial for subsequent treatment. Early examinations [4] relied on manually crafted features such as color and texture, but recent research based on deep learning methods has made polyp segmentation more accurate.

### 1.1. Research Progress on Polyp Segmentation

Traditional methods for polyp segmentation rely on manually created features, utilizing texture characteristics in images to capture the geometric orientation information of polyps [4,5,6,7,8,9,10,11]. Due to the irregular shapes, varying sizes, and different locations of polyps in colonoscopy images, these traditional methods have limitations, including false detection and poor generalization.

The emergence of Fully Convolutional Networks (FCNs) in 2015 [12] marked the beginning of research based on deep learning models. In 2017, the introduction of Transformer [13] led to the proposal of related Transformer-based models. By the end of 2023, the advent of Mamba [14] initiated research on polyp segmentation based on the Mamba model.

**Figure 1:**
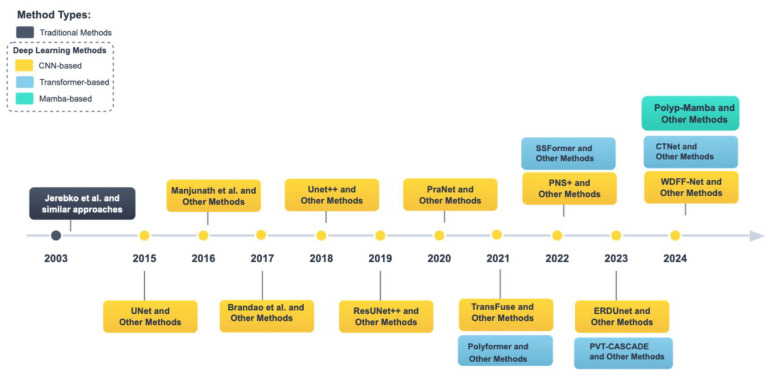
Progress in polyp segmentation research [6,15,16,17,18,19,20,21,22,23,24,25,26,27,28,29].

As shown in Figure 1, during the progress of polyp segmentation research, we selected Jerebko et al. [6] as a representative of traditional methods. For Convolutional Neural Network (CNN)-based models from 2015 to 2024, we chose UNet [15], Manjunath et al. [16], Brandao et al. [17], Unet++ [18], ResUNet++ [19], PraNet [20], TransFuse [21], PNS+ [22], ERDUnet [23], and WDFF-Net [24] as representatives for their respective years.

From 2021 to 2024, we selected Polyformer [25], SSFormer [26], PVT-CASCADE [27], and CTNet [28] as representative Transformer-based models for those years. We chose Polyp-Mamba [29] as the representative Mamba-based model for 2024.

### 1.2. Relevant Review Survey

Relevant research is shown in Table 1. The above review effectively summarizes existing polyp segmentation work but also has some shortcomings: Some reviews lack a systematic method summary table; some lack model performance evaluation analysis; some reviews analyze existing methods from a problem perspective, but with relatively few analyzed literature and papers. Compared to the review papers, this review encompasses 146 papers published between 2018 and 2024, representing both a longer time span and a larger corpus of analyzed literature, and additionally provides statistical analysis of video polyp segmentation methods. Second, the method and model section analyzes the advantages and disadvantages of method models; third, it classifies method models from the perspective of solving specific polyp segmentation problems and network architectures; fourth, the performance evaluation section provides performance analysis of these methods.

### 1.3. Our Contribution

This review aims to provide a comprehensive review of polyp segmentation methods, including CNN, Transformer, hybrid, and Mamba approaches for image-based segmentation as well as video polyp segmentation methods. It offers a systematic overview and summary, categorizing and analyzing each study based on the specific polyp segmentation problems addressed. Additionally, it evaluates each method in the performance assessment section.

By primarily collecting relevant literature from the past three years, this paper explores the main issues and corresponding solutions in current research in this field, providing a reliable reference for researchers. The main contributions of this review are as follows:

(1) Classify polyp segmentation methods into five categories based on their network architectures: CNN, Transformer, Hybrid, Mamba, and Other.
(2) Conduct a systematic study of polyp segmentation methods. Summarize the 87 papers and categorize the specific problems addressed by each polyp segmentation method into five categories.
(3) Provide a comprehensive compilation of datasets required for image and video polyp segmentation.
(4) We conducted a performance evaluation of methods for solving different problems, comparing and analyzing 44 models.
(5) Discuss the existing limitations and future research trends.

We organize the remaining sections of this review as follows: Section 2 introduces the literature search and review process. Section 3 defines polyps and discusses the challenges doctors face when managing them. Section 4 presents methods for polyp segmentation. Section 5 reviews the loss functions used in polyp segmentation. Section 6 describes the datasets used for polyp segmentation. Section 7 lists the metrics for evaluating models. Section 8 assesses the performance of these models. Section 9 explores the ongoing challenges in polyp segmentation. Section 10 investigates future research trends. Section 11 provides the conclusion.

## 2. Literature Search and Review Procedures

This review systematically assesses the effectiveness of deep learning methods for automated polyp segmentation in colonoscopy images. The primary research questions addressed are as follows: (1) How do various deep learning architectures—including convolutional neural networks (CNNs), Transformer-based models, hybrid approaches, and other emerging architectures—perform in colorectal polyp segmentation? (2) What are the current limitations associated with existing approaches, and what are promising avenues for future research?

Inclusion Criteria: Original research studies developing, validating, or evaluating deep learning models specifically for colorectal polyp segmentation were considered eligible. Studies included were published in English between 1 January 2018 and 10 December 2024; employed deep learning-based pixel-level segmentation techniques; reported at least one standardized segmentation evaluation metric (Dice coefficient or Intersection over Union); and were available as peer-reviewed journal articles or conference proceedings with accessible full texts.

Exclusion Criteria: The following types of articles were excluded: editorials, commentaries, or papers lacking full-text abstracts; studies relying exclusively on traditional image processing without deep learning; research focusing solely on polyp detection or classification without performing segmentation; studies lacking clear methodological descriptions or relevant performance metrics; duplicate publications; and non-English-language publications.

Information Sources and Search Date: We conducted a comprehensive search across Web of Science, IEEE Xplore, Scopus, and SpringerLink databases, employing the query combination: “polyp segmentation AND deep learning OR convolutional neural network”, further enhanced by including specific publicly available datasets (e.g., Kvasir, CVC-ClinicDB). The final literature search was completed on 10 December 2024.

**Figure 2:**
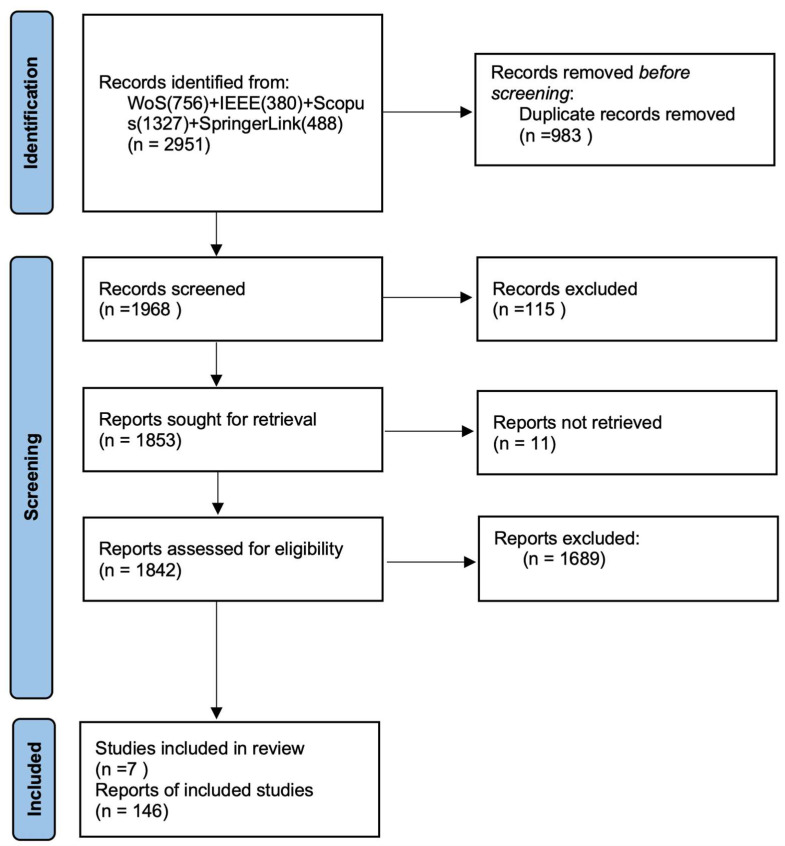
Flow diagram of the literature review procedures.

The initial database search identified a total of 2951 records across four electronic databases. After removing 983 duplicates, 1968 articles remained for further screening. Among these, 1689 papers were subsequently excluded based on the following criteria: (1) publications were limited to the past three years (2022–2024); (2) included studies were further assessed for eligibility based on the availability of source code links, citation counts exceeding 5, journal impact factors (IFs) greater than 3, clarity of methodological framework diagrams, and comprehensiveness of experimental validations on relevant polyp datasets. Ultimately, 146 original research articles met the inclusion criteria and were retained for detailed evaluation. The systematic literature review selection process is depicted in the PRISMA flow diagram (Figure 2) [37].

**Figure 3:**
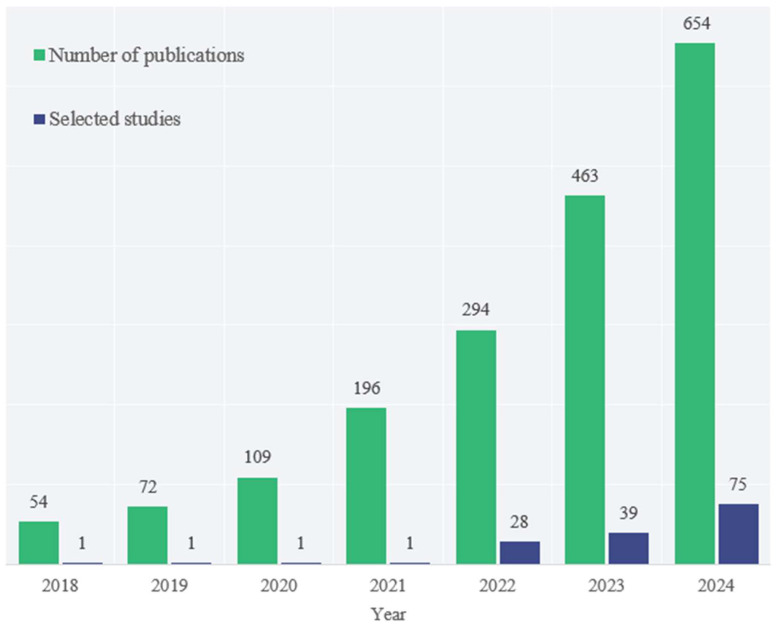
Distribution of published papers and selected papers from 2018–2024.

The screening criteria for papers from 2018–2021 include those with over a thousand citations in Google Scholar, as well as those with codes and over one hundred stars. The publication quantity of polyp segmentation research papers from 2018–2024 and their distribution after screening are shown in Figure 3.

## 3. Review About Polyp

In this section, we will explore three aspects: what polyps are, how doctors detect their presence, and the challenges doctors encounter when treating polyps.

**Figure 4:**
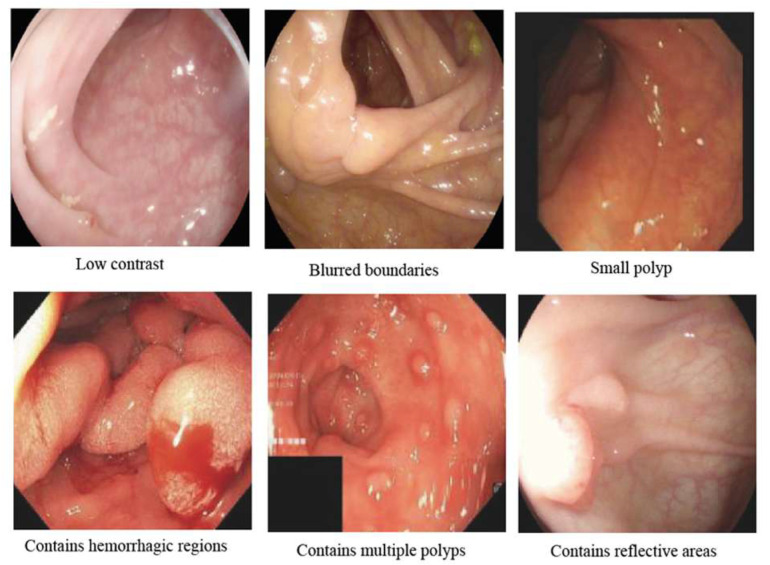
Diversity of polyp appearances across datasets. (**Top row** (left to right)): low contrast (ETIS-Larib), blurred boundaries (ETIS-Larib), small polyp (CVC-ClinicDB). (**Bottom row** (left to right)): contains hemorrhagic regions (Kvasir-SEG), contains multiple polyps (Kvasir-SEG), contains reflective areas (ETIS-Larib).

Polyps are abnormal tissue growths that protrude inward or outward from the mucosal surface; they typically appear mushroom-shaped or cauliflower-like and can range in size from a few millimeters to several centimeters [38,39]. The human body commonly finds them in various organs, especially the digestive tract [40]. Colon polyps grow on the inner walls of the colon or the rectum. Figure 4 shows the diversity of polyp appearances across datasets. Although most polyps are benign, tumorous polyps may carry a risk of becoming cancerous [41]. Approximately 70% of colorectal polyps are adenomatous polyps, which have the highest malignant potential, while hyperplastic polyps account for 10–30% and generally have low malignant risk [42,43]. Colon cancer survival rates vary depending on the stage of detection, ranging from over 95% in stage I to less than 35% in stages IV and V [44]. Therefore, timely detection and treatment are very important.

Doctors use ultrasound examinations or colonoscopies to check for the presence of polyps [45,46]. Colonoscopy is considered the gold standard for polyp detection, with a sensitivity of 75–93% for polyps ≥ 6 mm [47]. If doctors find polyps, they may recommend a biopsy and choose appropriate treatments based on the type and size of the polyps [48]. Screening and removal of colonic mucosal polyps are among the effective methods for preventing CRC [49]. Compared to the expected mortality rate in the general population, the CRC mortality rate in patients who had adenomas removed decreased by 53% within a median of 15.8 years [50]. Recent studies have shown that polypectomy can reduce CRC incidence by 76–90% [51].

**Figure 5:**
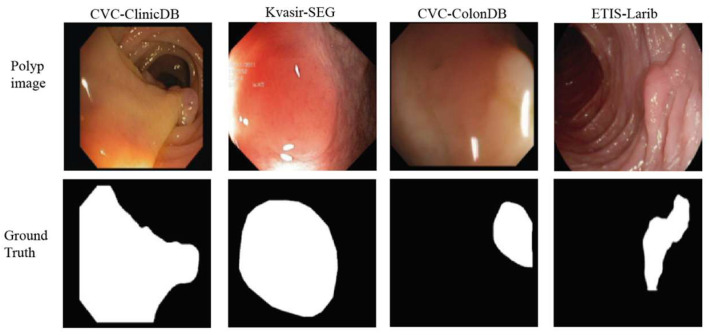
Image samples and corresponding segmentation masks from four benchmark datasets: CVC-ClinicDB, Kvasir-SEG, CVC-ColonDB, and ETIS-Larib.

Due to the varying types of polyps, complex morphologies, and indistinct polyp boundaries, doctors may encounter challenges in identifying polyps, particularly small or flat ones [52,53]. The miss rate for polyps during colonoscopy ranges from 6% to 27%, with higher rates for smaller polyps [54]. This can result in false positives or missed detections. Large polyps, polyps located in hard-to-reach areas such as bends or narrow sections of the intestine, and polyps growing near critical blood vessels are more challenging to segment and carry higher risks [55,56]. Different types of polyps, including adenomatous polyps and inflammatory polyps, have distinct histological characteristics, which can affect the difficulty and strategies for segmentation [57]. Doctors need to guarantee the complete removal of polyps and carry out comprehensive examinations. The quality of endoscopic images impacts physicians’ judgments and procedural accuracy [58]. Factors such as bowel preparation quality, withdrawal time, and endoscopist experience significantly affect polyp detection rates [59,60]. Blurry or unclear images may increase the difficulty and risk of surgery. The issues mentioned above require precise polyp segmentation techniques to assist doctors in handling them. Polyp segmentation is a pixel-level task that can accurately delineate polyps from colonoscopy images [61]. Figure 5 shows samples of polyp images from four datasets along with their corresponding segmentation masks. Continuous innovation and advancement in polyp segmentation models can significantly improve early diagnosis and precise treatment of polyps, thereby enhancing patients’ quality of life [62].

## 4. Polyp Segmentation Model

In this section, we categorize and describe methods based on network architectures, and within each architecture, we classify different approaches according to the specific problems they address. 

Currently, there are six major challenges in polyp segmentation: First, polyps vary in shape and size, exhibiting diversity in morphology, brightness, and color. This variability directly impacts two critical aspects: feature extraction becomes difficult due to the heterogeneous appearance of polyps, and boundary detection is challenged by blurred edges between polyps and surrounding tissue. Second, limited dataset issues arise from insufficient pixel-level annotations, making it difficult to train fully supervised polyp segmentation models. Small datasets can also lead to overfitting problems. Third, generalization performance remains a significant challenge, as trained models often show decreased performance on unseen test datasets. When the distributions of training and test data differ, domain adaptation techniques become necessary to address the decline in model performance. Fourth, real-time performance is crucial for clinical applications, where large parameter counts and computational complexity can hinder the deployment of models in practical endoscopy procedures. Fifth, low-quality frames in endoscopy videos, caused by factors such as motion blur, poor illumination, or artifacts, pose additional challenges for accurate segmentation. Finally, limitations of the architecture itself can constrain performance, as certain network designs may not fully capture the complex features required for precise polyp segmentation.

**Figure 6:**
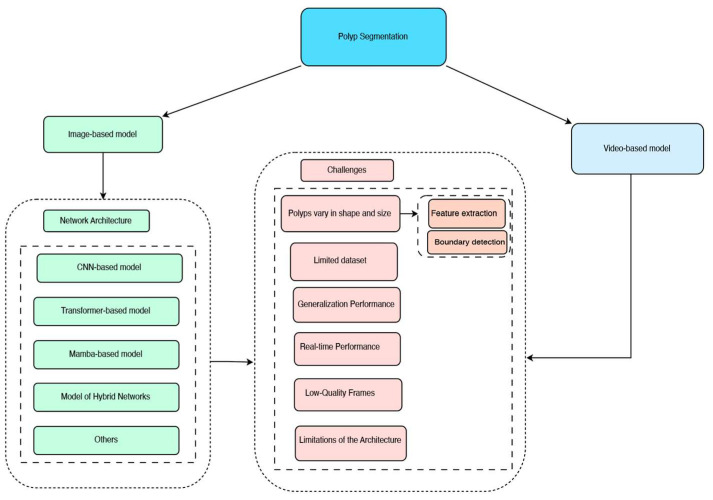
Classification of network architectures and challenges for polyp segmentation methods.

This section introduces polyp segmentation models based on images and videos from various network architectures. Figure 6 illustrates the classification of network architectures and the challenges faced by polyp segmentation methods. 

### 4.1. CNN-Based Methods

CNN-based polyp segmentation methods center around convolutional neural networks and often utilize architectures like U-Net and ResUNet. These methods perform multi-scale feature extraction and fusion by stacking convolutional and pooling layers. Skip connections are used to retain detailed information, making them well-suited for extracting local features. The advantage of CNN approaches lies in their high computational efficiency, but they may struggle to capture long-range dependencies, which can limit segmentation accuracy.

#### 4.1.1. Polyp Diversity and Boundary Factors

Addressing the diversity of polyps and the issue of blurred boundaries is crucial to the performance of polyp segmentation models. Therefore, this section reviews CNN-based models from two aspects: solving the problem of blurred boundaries and handling the morphological diversity of polyps.

##### Problems of Difficult Boundary Segmentation

The goal is to solve the difficult problem of precise boundary segmentation caused by unclear polyp boundaries. Bui et al. [63] proposed a multi-scale edge-guided attention network aimed at addressing segmentation challenges, such as the ambiguity of polyp boundaries and their varied morphologies. The method achieves fine edge enhancement by combining an encoder, a decoder, and an edge-guided attention module that uses the Laplacian operator. While this approach effectively highlights weak polyp boundaries and enhances segmentation performance, it requires further optimization for real-time performance. The goal of MISNet [64] is to solve the problems that come up when trying to separate polyps during a colonoscopy, such as changing lighting, polyps’ location and shape, and lines that are not clear. MISNet actively aggregates multi-scale features, generates guidance maps via a selective shared fusion module, and extracts and utilizes boundary information through a Parallel Attention Module and a Balanced Weight Module. This method effectively captures multi-level features, enhances boundary information extraction, and demonstrates excellent generalization ability on new datasets. However, the segmentation performance in extremely low-contrast environments still requires further improvement. Fan et al. [20] conducted a study to resolve the segmentation issue in endoscopic images, which arises due to the variability in polyp appearances and the lack of clear boundaries between them and the mucosa. They proposed the parallel reverse attention network as a solution. The system employs parallel part decoders to integrate high-level features into global maps, while a reverse attention module gradually eliminates estimated regions to unveil boundary details. This method effectively captures the polyp regions and boundary details. However, the model may still overlook precise constraints on polyp boundaries to some extent and has a significant reliance on high-quality annotated data. To solve the problems of appearance diversity and low boundary contrast in polyp segmentation, Liu et al. [65] came up with Focus on Boundary Segmentation (FoBS), a multi-level framework for improving the quality of segmentation boundaries. FoBS generates diverse polyp images through boundary-aware mixing, captures high-frequency boundary information with a variable Laplacian feature refinement module, and corrects degraded pixel regions using a position-sensitive compensation criterion. This framework synergistically enhances segmentation performance at the sample, feature, and optimization levels. It offers plug-and-play advantages, allowing for direct improvement in existing model performance, and is the first to extend the Laplacian operator into a deformable form for polyp segmentation. Its strengths lie in significantly enhancing boundary quality; however, it has limited effectiveness in segmenting atypical polyp morphologies and is sensitive to noise. CFA-Net proposed by Zhou et al. [66] addresses the challenges of polyps of various sizes and shapes, as well as the lack of clear boundaries with the surrounding environment. It integrates a boundary prediction network, a dual-stream segmentation network, and a boundary feature aggregation module to achieve cross-level feature fusion. This method effectively enhances segmentation performance for small polyps and in scenarios with ambiguous boundaries by combining boundary-aware features and multi-scale semantic information. Its strengths are particularly evident in situations with unclear boundaries; however, it does not meet real-time segmentation requirements and lacks the ability to capture fine boundaries for large polyps. Notably, the segmentation accuracy significantly decreases when the appearance of the polyp is similar to the background.

Yue et al. [67] addressed the issue of suboptimal polyp segmentation by proposing a boundary constraint network that integrates cross-layer context and edge information to enhance segmentation performance. This method utilizes a Cross-layer Feature Integration Strategy, which employs an attention mechanism to adaptively fuse high-level features, in conjunction with a Bilateral Boundary Extraction Module, which refines shallow polyp regions and non-polyp regions based on high-level positional information and boundary supervision. Its advantage lies in its excellent performance in situations with ambiguous boundaries and complex backgrounds. Nevertheless, in certain specific complex scenarios, BCNet may still exhibit inaccuracies in segmentation. Lin et al. [68] addressed the challenge of extracting boundary information in polyp segmentation by proposing the Bit-Slice Context Attention Network. This network includes the Bit-Slice Context Attention (BSCA) module, the Segmentation-Squeeze-Bottleneck Union (SSBU) module, the Multi-Path Attention Decoder (MCAD), and the Multi-Path Attention Encoder (MACE), which effectively suppress noise in feature maps and enhance feature representation. The BSCA module focuses on the boundary information between the polyp and surrounding tissue, while SSBU, MCAD, and MACE explore geometric information and detailed features from multiple perspectives. This method can effectively extract complex boundaries and improve segmentation accuracy. However, in scenarios where the background is highly similar to the polyp or where there are complex interferences, it may lose some low-level details, necessitating further robustness improvements.

##### Issues on Diversity in the Morphology, Size, Brightness, and Color of Polyps

To address the diversity in polyp shape, size, brightness, and color, Chen et al. [69] addressed the issue of color appearance imbalance and diverse morphologies leading to blurred boundaries in colorectal polyps with limited training data by proposing a method that combines Dataset-Level Color Augmentation (DLCA) and a Convolutional Multi-Scale Attention Module (CMAM). DLCA is based on dataset-level color statistics, significantly improving the color diversity of the training data and better addressing color imbalance. CMAM effectively handles the complexities of polyp size, shape, and boundary blurriness through multi-scale feature extraction and attention mechanisms. This method is the first to utilize dataset-level color statistics for data augmentation and deeply explores multi-scale contextual features. However, there is still a need for optimization in computational efficiency, and the issue of data scarcity remains to be resolved. UHA-Net [70] fully utilizes cross-layer and multi-scale features, aiming to address the challenge of scale variation in lesion areas within medical images. This framework includes a Hierarchical Feature Fusion module for coarse localization of targets, an Uncertainty Cross-layer Fusion module to enhance the effective integration of features from adjacent layers, and a Scale Aggregation Module that learns multi-scale features through convolutional kernels of different sizes. This approach excels in segmenting small-sized lesions and accurately locating lesion boundaries, effectively reducing both over-segmentation and under-segmentation issues. Conversely, its multiple-module design incurs a higher computational cost and shows a significant dependence on high-quality pixel-level annotated data. Wang et al. [71] addressed the issue of colon polyps being highly similar to their surrounding environment in terms of color, shape, and texture by proposing a semantic enhanced perception network. This network includes a multi-scale adaptive perception, module to enhance feature extraction, a polyp semantic enhancement module that utilizes coarse segmentation maps to extract semantic information, and a context representation calibration module that calibrates details through a branching network. SEPNet demonstrates excellent generalization ability in segmentation tasks within similar background scenes, achieving an inference speed of up to 62 Frames Per Second (FPS). However, it faces limitations in preserving edge details and has limited utilization of low-level local features. The FEGNet proposed by Jin et al. [72] aims to address the issue of ambiguous boundaries in polyp segmentation caused by variations in shape, size, and texture. The network employs a U-shaped architecture and integrates a feedback mechanism through a Recurrent Gate Module to optimize the attention maps of high-level features, while an edge extraction module is responsible for processing low-level features. The model enhances the learning process through three strategies: multi-scale supervision, recurrent supervision, and edge supervision. It performs exceptionally well in segmentation tasks involving complex shapes and smaller-sized polyps. However, its generalization capability still requires further improvement. CoInNet [73] aims to address the variability and unclear boundaries in polyps in terms of shape, color, size, and texture. This network combines the advantages of convolution and deconvolution operations, capturing the relationships between feature maps through statistical feature attention units and recursively fusing features using the Anomalous Boundary Approximation module to optimize segmentation results. It performs exceptionally well in the detection of small polyps. However, there remains a risk of missed detections and misclassifications when polyps coexist with folds or when there are interfering objects that resemble polyps. Wang et al. [74] addressed the challenge of segmenting small, low-contrast, and morphologically diverse polyps in colonoscopy images by proposing ISCNet, which integrates image-level and surrounding contextual information. This network includes a Global Guided Context Aggregation module and a Diversified Surrounding Context Focusing module, which enhance background–foreground differentiation and multi-scale feature extraction around the polyps, respectively. Through a combined loss strategy of binary cross-entropy and Dice coefficient, the model performs well in handling small polyps and demonstrates strong generalization capabilities. However, it may still encounter segmentation errors and misjudgments in scenarios where the polyp edges and surrounding textures are similar. Zhang et al. [75] proposed LDNet, which is based on a U-shaped structure, to address the diversity in polyps in terms of shape, size, and brightness, as well as the subtle issues related to background contrast. This network enhances the feature contrast between the polyps and the background region through lesion-aware cross-attention and captures long-range contextual relationships using efficient self-attention. The model improves segmentation accuracy by combining a dynamic kernel generation and updating mechanism with these attention-based approaches. However, the study did not delve into the model’s real-time performance and computational efficiency issues. Srivastava et al. [76] proposed a multi-scale residual fusion network to address the challenges of effectively segmenting multi-size targets and the difficulty of training on small-scale and biased datasets. This method introduces a Dual-Scale Dense Fusion block within the network to achieve cross-resolution feature exchange and employs residual dense blocks to reduce reliance on large training datasets. The Shape Stream and triple attention mechanism further enhance spatial accuracy and feature extraction capabilities while maintaining low computational complexity. However, the network’s performance in handling low-contrast images remains relatively weak. Liu et al. [77] addressed the issue that convolutional neural networks can only learn features in the spatial domain, making it difficult to capture more directional, singular, and regular patterns, by proposing a multi-scale contour-guided learning segmentation network. This method extends CNNs from the spatial domain to the spectral domain, obtaining multi-scale and multi-directional features through multi-scale contour sparse representation and enhancing spectral domain feature representation by utilizing contour knowledge to guide learning. Its advantage lies in achieving complementarity between the spatial and spectral domains, using contour kernels to make features more interpretable. However, the real-time performance of this network still needs improvement.

Some researchers propose using dual-branch network models to address challenges such as low polyp edge contrast and diverse polyp morphologies. Cao et al. [24] addressed the significant differences in color, size, shape, appearance, and location of polyps by proposing a weighted dual-branch feature fusion network (WDFF-Net) based on HarDNet68. This network enhances the segmentation performance of small and edge-blurred polyps through two complementary branches: Progressive Feature Fusion and Scale-Aware Feature Fusion, while also incorporating an Object-Aware Attention Mechanism. WDFF-Net demonstrates excellent generalization ability and segmentation performance when handling polyps of various morphologies. However, its drawback is that the inference speed is slightly lower than that of lightweight models such as HarDNet-MSEG. Song et al. [78] addressed the issues of significant variations in the shape and size of polyps, as well as the high similarity of small polyps to their surrounding environment, by proposing a multi-scale parallel network based on an attention mechanism. This network utilizes a multi-scale fusion module to integrate high-level features and comprehensively extract global and local information through parallel attention mechanisms, while optimizing edge details using reverse context fusion. Its advantage lies in balancing features of different scales and significantly improving the segmentation performance of small polyps. However, the model’s performance still needs enhancement when faced with complex real-world scenarios, such as liquid occlusion.

To solve the problem of difficult precise segmentation of small polyps, Liu et al. [79] addressed the issue of insufficient boundary detail and global context information extraction in the segmentation of small polyps by proposing the FTMF-Net model. This model extracts fine boundary features from the frequency domain using a Fourier Transform (FT) module and enhances high-level semantic information on the encoder side through a Multi-Scale Feature Fusion module. This is the first application of the Fourier Transform in small polyp segmentation, significantly strengthening the ability to capture subtle boundaries. However, when faced with complex and difficult samples, the model’s segmentation performance still requires further improvement.

#### 4.1.2. Limited Polyp Dataset

Due to limited annotated datasets, some researchers use semi-supervised models for polyp segmentation. To address the challenges posed by the reliance on large amounts of labeled data in fully supervised methods, as well as the variations in shape, size, and location of lesions, Gu et al. [80] proposed the semi-supervised segmentation framework DEC-Seg. This framework uses cross-layer feature aggregation, a scale-enhanced consistency strategy, and a dual-scale complementary fusion module to get the most out of the small amount of labeled data that is available. The model outperformed existing semi-supervised methods with only 10% and 30% labeled data. However, it still has certain limitations when dealing with ambiguous regions and small lesions in the background. Ren et al. [81] proposed the WS-DefSegNet framework, which combines weak supervision and semi-supervision to reduce the strong reliance on pixel-level annotations for polyp segmentation. This framework utilizes a newly constructed weakly labeled dataset, W-Polyp, and employs a sparse foreground loss function to suppress false positives during weakly supervised training while enhancing features with a deformable Transformer encoder. It can complete training with only 1.9% of pixel annotations, significantly reducing the annotation burden on physicians, and demonstrates good transferability. However, the mean Dice coefficient (mDice) score of approximately 0.768 on datasets like Kvasir still falls short of the fully supervised method PraNet, which achieves a score of 0.898.

Yu et al. [82] addressed the issues of overfitting in small-scale datasets and the high computational complexity of Transformer by proposing a novel non-pretrained deep supervision network called NPD-Net. To avoid overfitting, it uses a non-pretrained deep supervision strategy. The parallel dual attention module combines spatial and channel attention to make the computer work faster while still being able to model features. Compared to most advanced methods, NPD-Net has fewer parameters and can significantly reduce overfitting on small datasets. However, its segmentation performance still falls short when dealing with ambiguous images or those containing bubbles.

Guo et al. [83] proposed a method that combines meta-learning mixed data augmentation and confidence-aware resampling strategies to address the issues of limited training data, significant variations in polyps, and class imbalance. This method utilizes meta-learning to acquire clinical diagnostic knowledge from validation data, generating compatible hard labels for mixed images, and employs a progressive resampling strategy that moves from easy to difficult at both the image and pixel levels to balance the data distribution. It achieved good segmentation results in low-contrast and varying lighting conditions. However, its performance in extremely imbalanced scenarios has not been thoroughly discussed, and real-time performance has not been evaluated in detail.

Wang et al. [84] addressed the issue of high costs associated with dense annotations and the limitations of existing weakly supervised methods that are confined to spatial domain learning by proposing the S^2^ME segmentation framework based on doodle supervision. This framework performs feature learning in both spatial and spectral domains, generating reliable mixed pseudo-labels by combining an entropy-guided pseudo-label ensemble learning strategy with adaptive pixel-level fusion techniques. High-quality segmentation results can be achieved solely by relying on doodle annotations. However, this method still lacks comparisons with the latest approaches.

#### 4.1.3. Generalization of Training Models and Domain Adaptation

To enhance the model’s generalization performance, Jia et al. [85] focus on the challenges of limited labeled data and class imbalance in polyp segmentation, proposing the PolypMixNet semi-supervised framework. This framework enhances the utilization of unlabeled data through polyp-aware augmentation techniques and dual consistency regularization under the Mean Teacher structure. It achieves near-full-supervised performance with only 15% labeled data but still performs worse than fully supervised methods on smaller datasets such as CVC-ColonDB and ETIS-Larib. Zhang et al. [86] addressed the issue of decreased polyp segmentation performance caused by domain transfer by proposing a domain generalization method based on random global illumination enhancement. This method utilizes an Image Decomposition Module to decompose images into three components: reflectance, local illumination, and global illumination. It then employs an Illumination Transformation Module to generate global illumination in the style of the target domain, thereby increasing data diversity. This method can effectively segment polyps under complex shapes and various lighting conditions. However, there are still shortcomings in handling other domain shift factors, such as color and shape differences.

Lu et al. [87] addressed the issue of decreased polyp segmentation performance caused by distribution differences between the source and target domains by proposing the DCL-PS cross-domain polyp segmentation framework. This framework achieves cross-domain feature alignment through Domain Contrastive Learning (DCL) and Contrastive Learning with Cross-Consistency Training and utilizes a Prototype-guided Self-training strategy to dynamically assign weights to pixels. It demonstrates excellent capability in cross-domain feature alignment. However, there are still shortcomings in capturing edge details of large polyps. Wang and Chen [88] proposed the RPANet unsupervised domain adaptation framework to address the issue of polyp segmentation transfer learning in scenarios where source domain data cannot be obtained. This method employs two modules: foreground-aware contrastive learning and confidence-calibrated pseudo-labeling, to achieve efficient adaptation of the model by self-supervised learning of target domain data features from coarse to fine. It does not require labeled data from the source domain, meeting the privacy and cross-hospital deployment needs of medical scenarios. However, there are still limitations in processing when the differences between foreground and background are not significant, and the issues of noise and errors accumulated in the pseudo-labels have not been fully resolved.

#### 4.1.4. Real-Time Performance of the Model

To improve real-time performance, maintain a smaller model parameter count, and enhance computational efficiency, Li et al. [23] addressed the challenges of extracting global contextual features and the large model parameters that hinder clinical applications by proposing the ERDUnet segmentation network. This network achieves high precision and lightweight segmentation through modules such as the Context Enhanced Encoder and Differential Regional Attention, as well as a dual U-shaped structure. While maintaining a small parameter size and fast inference speed, ERDUnet can accurately handle large-scale targets. However, when the features of the target and background are too similar, the model is still prone to recognition errors.

### 4.2. Transformer-Based Methods

Transformer-based polyp segmentation methods primarily leverage the self-attention mechanism in Transformer to model global information, thereby better understanding the overall shape and context of polyps. Examples of such architectures include TransUNet and Swin-UNet. Transformers are excellent at capturing long-range dependencies, which can enhance the accuracy and stability of segmentation in complex scenarios. 

#### 4.2.1. Polyp Diversity and Boundary Factors

The model needs to have strong feature extraction capabilities to handle the morphological diversity of polyps and sensitivity to boundary information. We review Transformer-based models from these two perspectives.

1. Issues on Diversity in the Morphology, Size, Brightness, and Color of Polyps

To address the challenges of existing methods in capturing high-level semantics and dealing with the camouflage characteristics of polyps, Xiao et al. [28] proposed a novel polyp segmentation framework named CTNet. This framework trains a Transformer backbone network through supervised contrastive learning and incorporates a self-multi-scale interaction module and an information collection module to extract structured features and multi-scale information. Experimental results show that CTNet improves mDic and mean Intersection over Union (mIoU) by 1.5% and 1.6%, respectively, compared to the second-best method, ColonFormer-L. However, it has a high dependency on pixel-level annotated data, and there is still room for improvement in the segmentation performance of small target polyps. Wu et al. [89] addressed the problem of segmenting polyps that are morphologically diverse and difficult to distinguish from the surrounding mucosa by proposing the MSRAformer multi-scale spatial reverse attention network. This method uses a pyramid-structured Swin Transformer as the encoder and combines multi-scale channel attention modules with a spatial reverse attention mechanism to progressively enrich and refine the edge structure and details of the polyp region. The use of circular residual convolution blocks enhances feature learning capabilities and effectively prevents gradient vanishing. The model demonstrates excellent generalization ability in extracting and aggregating multi-scale features, but there is still room for improvement in segmenting tiny lesion areas. Liu et al. [90] proposed the MLFF-Net multi-layer feature fusion network to address the issues of insufficient feature utilization and semantic conflicts arising from feature fusion in existing methods. This network works collaboratively through a multi-scale attention module a high-level feature enhancement module, and a global attention module to integrate shallow details with deep semantic features. It has achieved high accuracy and robustness in segmenting small or irregularly shaped polyps. However, it still has certain limitations when handling the segmentation of multiple polyps. Xu et al. [91] came up with PSTNet, which combines RGB and frequency domain information to improve the accuracy of colon polyp segmentation. This was done because previous methods relied too heavily on RGB information and misaligned features, which made it hard to accurately identify polyps. This network includes a frequency feature attention module, a feature supplement alignment module, and a cross-perception localization module, which extract low-contrast polyp cues from the frequency domain and reduce misalignment interference during multi-scale feature fusion. Compared to existing methods, PSTNet demonstrates improved detection capabilities for small polyps and edge regions while effectively suppressing noise. However, there remains a need for improvements in computational efficiency. Yue et al. [92] addressed the challenges of polyp segmentation in complex backgrounds, varying shapes and sizes, and fuzzy boundaries by proposing the Progressive Feature Enhancement Network. This network employs a pyramid vision Transformer as the encoder and integrates three modules: Cross-Stage Feature Enhancement, Coarse Map Generation, and Foreground Boundary Collaborative Enhancement, enabling the network to progressively refine segmentation results from coarse to fine. By eliminating redundant information and enhancing multi-scale feature interaction, PFENet demonstrates a stronger feature representation capability in recognizing complex backgrounds and deformed polyps. However, when dealing with data from different domains, its generalization performance still requires further improvement. Dong et al. [93] addressed the limitations of CNN backbones in cross-layer feature fusion by proposing the Polyp-PVT framework, which utilizes a Pyramid Vision Transformer to obtain more robust feature representations instead of traditional CNNs. This method includes a Cascade Fusion Module, a Camouflage Identification Module, and a Similarity Aggregation Module, which are used for high-level semantic fusion, low-level camouflage feature identification, and cross-layer feature integration, respectively. It performs exceptionally well in tasks such as video polyp segmentation, particularly surpassing the existing method PNS-Net in feature extraction and generalization capabilities. However, the model’s detection of polyp boundaries still needs improvement in complex areas with overlapping light and shadow. Liu et al. [94] proposed the CAFE-Net segmentation network to address issues such as the loss of small-sized targets, difficulty in recovering fine-grained details, and insufficient multi-scale feature aggregation. This network compensates for missing details and explores potential features through the Feature Supplementation and Exploration Module, retains low-level information and recovers fine-grained features using the Cross-Attention Decoder Module, and maximizes the utilization of learned features with the Multi-Scale Feature Aggregation Module. It demonstrates excellent performance in handling small-sized polyps, complex lighting, and blurred boundaries. However, potential overfitting and computational efficiency require further improvements. Li et al. [95] proposed the Lesion-Aware Context Interaction Network to address the issues of diverse foreground shapes, complex background interference, and high computational costs. This network reduces background noise through a Lesion-Aware Pyramid Mechanism and enhances global feature representation by combining a Non-Local Context Lesion Interaction Module in the Robust Representation Enhanced Decoder and a Triplet-Branch Multi-Scale Perceptual Self-Attention Module. LACINet demonstrates excellent segmentation performance for diverse polyp morphologies while maintaining real-time capability at 28.41 fps. However, the segmentation accuracy for tiny polyps and under extreme lighting conditions still needs improvement.

2. Problems of Difficult Boundary Segmentation

To solve the problem of difficult precise boundary segmentation caused by unclear polyp boundaries, Cai et al. [96] proposed VANet to address the diversity of polyp appearances and the issue of blurred boundaries caused by changes in the endoscopic viewpoint. This network locates polyp regions through a viewpoint classifier’s class activation map and enhances polyp feature representation and optimizes segmentation boundaries by combining viewpoint-aware Transformer and boundary-aware Transformer. The method guides attention by using viewpoint information to cut down on interference from areas that are not polyps and improves boundary segmentation for polyps even more through multi-scale predictions. VANet adapts well to changes in viewpoint and improves edge recognition accuracy. However, the model has a large number of parameters, and its performance significantly declines when handling very small or very large polyps.

#### 4.2.2. Limited Polyp Dataset

Rahman et al. [97] proposed the PP-SAM perturbation prompt method to address the Segment Anything Model’s (SAM) reliance on a large amount of labeled data when fine-tuning on new data sources. This method enhances the model’s adaptability to imprecise prompts by introducing variable bounding box prompt perturbations during the fine-tuning process, only fine-tuning the image encoder and prompt encoder while freezing the mask decoder. On the Kvasir dataset, using bounding box prompt perturbations of 50 and 100 pixels improved the DICE scores by 20% and 37%, respectively, demonstrating excellent few-shot learning capabilities. However, when the sample size exceeded 50, the performance improvement was no longer significant.

#### 4.2.3. Generalization of Training Models and Domain Adaptation

To address the generalization and detail-handling challenges of deep learning models in polyp segmentation tasks, Wang et al. [26] addressed the challenges of complex polyp morphology and model overfitting by proposing the SSFormer model to enhance generalization ability and detail processing. This model combines a pyramid Transformer encoder with a Progressive Local Decoder and enhances local detail processing capabilities through Local Emphasis and Stepwise Feature Aggregation modules. SSFormer demonstrates strong generalization ability and local feature processing capability across different polyp datasets through multi-stage feature aggregation and progressive feature fusion. However, the model’s performance on extreme or unseen medical images still needs validation, and it requires a long training cycle with high demands on the quality and quantity of training data.

#### 4.2.4. Real-Time Performance of the Model

To improve real-time performance, maintain a smaller model parameter count, and enhance computational efficiency, Jha et al. [98] proposed the Transformer-based Residual Upsampling Network to meet the demand for automatic real-time polyp segmentation. This network effectively avoids over-segmentation and under-segmentation issues while maintaining a real-time processing speed of 47.07 FPS. However, its performance still shows a significant decline on out-of-distribution datasets, indicating certain limitations in generalization. Lin et al. [3] addressed the issues of high computational complexity and large parameter size in existing Vision Transformer (ViT) models for polyp segmentation by proposing a lightweight visual Transformer model called Polyp-LVT. This method significantly reduces the model’s computational complexity and parameter count by replacing the attention layer with a global max pooling layer in the encoder, and it incorporates the IFFM module in the decoder for efficient feature fusion. Polyp-LVT reduces the parameters by approximately 44% compared to the baseline model Polyp-PVT, with a computational requirement of only 13.21 Giga Floating Point Operations Per Second (GFlops). However, there is still room for further improvement in model accuracy.

#### 4.2.5. Limitations of Architecture

Huang et al. [99] addressed the issue of feature information loss caused by patch segmentation in Transformer models by proposing the HST-MRF model, which integrates multi-scale receptive fields. This model combines the Heterogeneous Swin Transformer with a multi-modal bilinear pooling module to effectively integrate features from different receptive fields while also incorporating both low-level and high-level semantic information for more accurate lesion localization. This approach maintains high segmentation performance while reducing computational complexity and effectively minimizing the loss of structural information. However, the precision metric still shows shortcomings, particularly in the recognition of lesion boundaries, which requires further improvement.

Lin et al. [100] addressed the issue of the Transformer block division neglecting pixel-level intrinsic structural features by proposing DS-TransUNet, which incorporates a hierarchical Swin Transformer into the encoder and decoder of a U-shaped architecture. Through a dual-scale encoding mechanism, the model simultaneously captures coarse and fine-grained features and utilizes self-attention to model non-local dependencies and multi-scale contextual information. This strategy effectively enhances the semantic segmentation quality of medical images. However, when both encoding branches use the same patch size, the model’s performance still has certain limitations. Rahman and Marculescu [27] proposed the CASCADE attention decoder to better capture local and long-range dependencies between pixels in medical images based on the Transformer architecture. They suppressed background interference and enhanced detail representation through a cascade design of multi-scale features, attention gates, and convolutional attention modules. A multi-stage feature and loss aggregation framework further improved the accuracy and convergence speed of segmentation. However, the model still requires more experimental validation to demonstrate its robustness in terms of generalization capability.

### 4.3. Hybrid Architecture Methods

A polyp segmentation method that combines the CNN and Transformer leverages the CNN’s strength in capturing local features and the Transformer’s ability to model global features. Typically, CNNs are used to extract basic features in the early or middle stages of the network, and Transformer modules are integrated later or concurrently for global self-attention learning. This approach can significantly enhance the accuracy and robustness of segmentation.

#### 4.3.1. Polyp Diversity and Boundary Factors

Blurred boundaries increase the difficulty of effective feature extraction, and challenges arising from the natural characteristics of polyps affect polyp segmentation performance. In this subsection, we will review how hybrid polyp segmentation methods based on CNNs and Transformer address these two issues.

1. Issues on Diversity in the Morphology, Size, Brightness, and Color of Polyps

To address the diversity in polyp shape, size, brightness, and color, Zhang et al. [21] proposed the parallel branch architecture TransFuse to simultaneously address the modeling of local spatial details by CNNs and global dependencies by Transformer. This method efficiently fuses multi-level features between parallel branches using an innovative BiFusion module that leverages self-attention and bilinear Hadamard products. While maintaining a low parameter count, TransFuse-S achieves an inference speed of 98.7 FPS. However, the study has not sufficiently validated its capability to handle tiny objects and extremely imbalanced datasets.

Liu et al. [101] proposed MVOA-Net to tackle intra-class confusion stemming from inconsistencies in polyp morphology, size, and location, as well as inter-class indistinguishability resulting from high similarity with surrounding tissues. This model uses a Geometric Orientation Transformer Encoder to pull out features from both horizontal and vertical views. It also uses a Point Affinity Module to improve boundary detection and a Cross-Attention Fusion Module to combine global and local data. In multi-target polyp segmentation, MVOA-Net does a great job in terms of accuracy and parameter efficiency. It has great geometric orientation and point perception skills. However, its computational efficiency still requires further improvement. The Region Self-Attention Enhanced Network was created by Yin et al. [102] to solve the problem of segmentation that arises from the high similarity between the edges of normal tissue and polyps. This network is built on Pyramid Vision Transformer v2 (PVTv2) and makes different feature maps using a dual-decoder structure. It does this by using the Region Self-Attention (RSA) module to obtain more accurate boundary information. The design of the dual decoders combined with RSA accommodates both small and normal-sized polyps, significantly improving segmentation performance. However, the model still faces certain limitations in effectiveness when dealing with complex samples that have reflections and shadows. Yang and Zhang [103] proposed PFD-Net to better capture local and global semantic information in medical images. This network combines the PVTv2 Transformer with a CNN encoder and utilizes fast Fourier convolution and deformable convolution modules. The network becomes better at showing complicated shapes and uneven edges. This leads to clearer target boundary predictions and richer semantic structure extraction. However, the model’s segmentation performance remains unstable in scenarios where the target and background are extremely similar or occluded. To fix the problems of multi-level semantic feature gaps and weak connections between spatial and channel dimensions, Wu et al. [104] suggested a hierarchical mixed architecture they called HD-Former. This model uses a Compression Bottleneck module to improve shallow features and find target areas. It also includes a Dual Cross-Attention Transformer module for flexible fusion of multi-scale features and a Broad Exploration Network for hierarchical dense contextual semantic capture. This approach preserves detailed information while bridging the differences between various semantic levels on a global scale. However, the increased depth of the backbone network significantly raises the computational load. To address the issue of diverse lesion types in medical images that are similar to surrounding tissues, Du et al. [105] proposed the SwinPA-Net. The Dense Multiplicative Feature Fusion module minimizes interference from shallow background noise, while the Local Pyramid Attention module combines global and local attention to guide the network’s focus on the lesion areas. This innovative design provides strong generalization capabilities, but the large number of model parameters still limits its real-time performance. Zhang et al. [106] suggested HSNet, which combines Transformer and a CNN, as a way to fix the problems of polyps changing sizes, shapes, and lighting, as well as the lack of attention to fine textures. It combines long-range and local features through the Cross-Semantic Attention module and the Hybrid Semantic Complement module and utilizes the Multi-Scale Prediction module to adaptively fuse outputs of different scales. This method takes into account both global appearance and detailed textures, significantly enhancing segmentation performance. However, the detection of tiny target polyps still requires further improvements.

Li et al. [107] proposed FMCA-Net to address issues such as inconsistencies within polyps and similarities with surrounding tissues in medical image segmentation. This network uses PVTv2 as the encoder and further explores the initially extracted features through a feature secondary reuse module, supplemented by a group of dilated convolution attention modules to enhance edge segmentation. This approach effectively utilizes early polyp features while improving the problem of blurred segmentation boundaries. However, there is still room for improvement in the model’s performance in scenarios with densely distributed or very small polyps.

Jiang et al. [108] addressed the issue of the overly complex structure of Transformer and the insufficient focus on decoders by proposing the MLP-CNN dual-path complementary network. This network replaces the traditional Transformer with a Multi-Layer Perceptron (MLP), using dual-path decoders to separately reconstruct global and local information. It employs a Dual-Path Complementary (DPC) module to fuse multi-layer features from both MLP and CNNs, ultimately merging the segmentation results through the segmentation mask feature fusion module. The DPC module performs well in multi-scale and fuzzy boundary scenarios while also being parameter-efficient and offering higher training and inference efficiency. However, in complex scenarios, the model still has a significant demand for large-scale data, and its generalization ability requires further validation. Wu et al. [109] addressed the issue of high computational costs and insufficient detail feature learning in traditional Vision Transformer for polyp segmentation by proposing META-Unet. This network introduces a multi-scale efficient Transformer attention mechanism within a U-shaped architecture, generating element-level attention maps through a dual-branch structure that captures features at different scales effectively. This design allows for simultaneous modeling of global context and local details, significantly improving the accuracy and efficiency of segmentation. However, the model’s segmentation performance remains suboptimal for very small, very large, or poorly defined boundary polyps. Sanderson and Matuszewski [110] proposed FCBFormer to address the issues of overfitting in deep learning for segmentation and insufficient resolution in Transformer. This network extracts key features through a transformer branch while utilizing a fully convolutional branch and its dense skip connections to ensure boundary accuracy. Its advantage lies in simultaneously leveraging Transformer for global feature extraction and the CNN for maintaining segmentation details. However, the model still has a strong dependence on training data.

2. Problems of Difficult Boundary Segmentation

To solve the problem of difficult precise boundary segmentation caused by unclear polyp boundaries. Li et al. [111] addressed the issue of significant internal differences in polyps and the ambiguous boundaries with surrounding tissues by proposing a Cross-layer Information Fusion and Guidance Network. This network extracts multi-scale features through a Transformer encoder. On the CVC-ClinicDB dataset, it improved mDice by 3.9% and mIoU by 4.2% compared to PraNet, demonstrating greater robustness against interferences such as lighting reflections. However, this method did not discuss the limitations related to inference speed and resource consumption in practical applications in detail. Shao et al. [112] proposed the Polyper method to address the segmentation problem of small polyps and ambiguous boundaries. This method draws on clinical experience to extract boundaries and polyp regions using morphological operators. It employs a boundary-sensitive attention mechanism to feedback the features of the internal polyp region to the boundary area, enhancing segmentation accuracy. The method performs well in scenarios involving small polyps and ambiguous boundaries. However, its fixed boundary width design has limitations in handling false positives and false negatives, making it challenging to adequately address the diverse characteristics of polyps. To solve the problems of Transformer not being able to extract enough local features and the CNN having trouble modeling long-range relationships, Cai et al. [113] came up with PPFormer, which combines Transformer’s long-range modeling skills with the CNN’s local feature extraction skills. The PP-guided self-attention mechanism focuses on hard-to-segment areas and utilizes a local-to-global mechanism to obtain more local information while maintaining low memory usage, achieving more accurate polyp localization. This method significantly enhances the perception of polyp boundaries. However, the segmentation performance on small-sized polyps still requires further improvement.

#### 4.3.2. Limited Polyp Dataset

To address the need for reducing the annotation cost of medical image segmentation while maintaining accuracy, Xie et al. [114] proposed SimTxtSeg, a weakly supervised method. This method utilizes a text-to-visual cue converter and a text-visual hybrid attention module to train the segmentation model using simple text prompts and pseudo-labels generated by SAM. Compared to traditional fully supervised methods, SimTxtSeg significantly reduces annotation costs and demonstrates the effectiveness of cross-modal feature fusion. However, the overall performance may suffer if the quality of the pseudo-masks generated by the SAM model is insufficient.

#### 4.3.3. Generalization of Training Models and Domain Adaptation

Li et al. [115] proposed the ASPS method to address the issues of insufficient local detail capture and poor out-of-distribution performance in the SAM model for endoscopic images. This method integrates CNN and ViT encoder features through a cross-branch feature augmentation module, enhancing the capture of local details and high-frequency information. Meanwhile, the uncertainty-guided prediction regularization module reduces prediction bias and improves the generalization ability of out-of-domain segmentation. However, there is still room for further optimization of ASPS in the segmentation of small target polyps. Xiong et al. [116] proposed SAM2-UNet, aiming to create a universal segmentation framework that is applicable to both natural images and medical images. This method is based on the Hiera network of SAM2 as the encoder, incorporating an adapter for parameter-efficient fine-tuning, combined with a classic U-Net decoder. Its advantages include being more friendly to devices with limited memory while demonstrating excellent performance in medical image segmentation. However, its performance on specific datasets, such as ClinicDB, still lags behind some other advanced methods.

#### 4.3.4. Real-Time Performance of the Model

Jha et al. [117] proposed TransNetR to address the shortcomings of existing automated segmentation methods in terms of real-time processing speed and generalization ability. The encoder is a pre-trained ResNet50, and the self-attention mechanism of the Residual Transformer module improves the decoder’s ability to learn features. It achieves high-precision segmentation on polyps of various sizes and shapes, with a processing speed of up to 54.60 FPS. However, the recall metric is slightly lower than that of UACANet on certain specific center data, indicating room for improvement in the identification of very small polyps.

### 4.4. Mamba-Based Methods

The polyp segmentation method based on Mamba primarily leverages Mamba’s automatic segmentation capabilities and optimization algorithms to achieve fast and accurate segmentation. By employing a selective State Space Model (SSM), it can handle long sequence dependencies with linear complexity, offering greater efficiency than Transformer.

#### 4.4.1. Polyp Diversity and Boundary Factors

Some researchers have employed Mamba-based methods to address the diversity in polyp morphology and the issue of blurred boundaries. We will introduce these methods in this section.

1. Issues on Diversity in the Morphology, Size, Brightness, and Color of Polyps

Xie et al. [118] addressed the problem of segmenting polyps with diverse morphologies and unclear boundaries by proposing the Prompt-Mamba method, which combines Vision-Mamba with box-prompt. This model consists of three components: a lightweight image encoder, a prompt encoder, and a mask decoder. It introduces bidirectional sequence modeling and a reverse SSM branch to better capture image features. The use of box-prompt enhances the model’s stability and generalization, while the combination of Focal Loss and Dice Loss improves segmentation quality. However, its performance on certain datasets is currently slightly lower than that of existing state-of-the-art methods, indicating that there is still room for improvement in generalization capability. Zhang et al. [119] addressed the issues of insufficient long-range dependency modeling in CNNs and the high computational complexity of Transformer by proposing the VM-UNetV2 based on SSM. This model introduces a Visual State Space (VSS) block to capture global contextual information and employs a semantic detail integration module to enhance cross-layer feature fusion. Compared to traditional CNNs and Transformer, VM-UNetV2 makes breakthroughs in both long-range dependencies and computational complexity while also improving segmentation performance through deep supervision. However, its robustness and generalization capabilities still require further empirical validation. Xu et al. [29] addressed the limitations of existing medical image segmentation models in long-range dependencies and cross-scale feature interactions by proposing the Polyp-Mamba framework. By introducing the Scale-Aware Semantic module and the Global Semantic Injection module, this model effectively captures multi-scale semantic information and enhances local features while maintaining linear computational complexity. It demonstrates excellent segmentation capabilities for polyps of various sizes, shapes, and textures. However, the model requires a high number of training iterations, and its interpretability still needs further improvement. Dutta et al. [120] proposed the SAM-Mamba architecture, aimed at addressing issues such as the variability in polyp shapes, ambiguous boundaries, and difficulties in generalization on unseen datasets. The SAM-Mamba method, based on SAM, achieves efficient polyp segmentation by introducing the Mamba-Prior module into the architecture and employing a multi-scale spatial decomposition and two-stage training strategy. Its advantages include excellent zero-shot generalization capability, multi-scale feature capture, and stable performance even under complex boundaries and textures. The main drawbacks are that edge detection accuracy still needs improvement, there may be misjudgments in complex scenes, and there is the computational burden brought by high-resolution inputs of SAM.

2. Problems of Difficult Boundary Segmentation

Zhu et al. [121] came up with the Hybrid Multi-Frequency Perception Gated Selection Network Polyp-Mamba to deal with problems like unclear polyp boundaries and a significant number of similarities with nearby tissues. This model extracts global and local features through a dual multi-frequency fusion encoder combining Mamba and ResNet and enhances the boundaries of ambiguous regions using multi-frequency spectrum analysis via Discrete Cosine Transform (DCT). In the decoding phase, the gated selection decoder can suppress irrelevant areas and improve segmentation accuracy, while deep supervision further ensures effective alignment between features and labels. Although this method performs excellently across multiple datasets and approaches real-time processing, it may misjudge in certain scenarios with clear boundaries, and DCT still has limitations in capturing details within complex structures.

#### 4.4.2. Limitations of Architecture

Wu et al. [122] addressed the challenges of CNNs in processing long sequence information and the low sensitivity of Vision Transformer to local features, as well as the drawback of the SS2D module introducing redundant information while maintaining a global receptive field. They proposed a high-order visual Mamba UNet based on an SSM. By utilizing a high-order two-dimensional selective scanning module, they reduced redundant information while preserving the advantages of a global receptive field, in conjunction with a Local-SS2D module to enhance local feature representation. This method integrates H-SS2D into a high-order visual state space module, achieving efficient medical image segmentation. However, the model currently lacks a systematic analysis and validation of its robustness.

### 4.5. Other Architectural Methods

There are also models that do not belong to the above four categories of architecture, such as architectures based on diffusion probabilistic models, models based on the Broad Learning System, and the Segment Anything Model. The emergence of these models has also expanded research in the field of polyp segmentation.

#### 4.5.1. Polyp Diversity and Boundary Factors

Some researchers have used diffusion probability models and the general segmentation model SAM to address the diversity of polyps and the issue of blurred boundaries in polyp segmentation.

1. Issues on Diversity in the Morphology, Size, Brightness, and Color of Polyps

Du et al. [123] addressed the challenges of diverse polyp morphology, data scarcity, and poor contrast with surrounding tissues by proposing the Highlighted Diffusion Model (HDM) and the HDM+ framework. This method uses a two-step training process. first, it trains the HDM to highlight polyp regions while suppressing the background, and then uses the HDM features as prior knowledge for polyp segmentation, achieving feature fusion through a cross-attention mechanism. This design reduces the domain gap between RGB images and segmentation masks while also enhancing segmentation accuracy and efficiency. However, there are still certain challenges in segmenting very small or poorly defined polyps.

2. Problems of Difficult Boundary Segmentation

Wang et al. [124] focused on improving the efficiency and accuracy of colorectal polyp segmentation, proposing an end-to-end segmentation method based on a Denoising Diffusion Probabilistic Model (DDPM). This method uses U-Net as the backbone, cascading the original image with a noise mask to learn their intrinsic relationship, and generates more precise segmentation results through multiple resampling and majority voting strategies. This is the first application of DDPM in the field of polyp segmentation, effectively handling images of polyps with blurred boundaries. However, the model’s generalization performance still requires further validation and enhancement.

#### 4.5.2. Limited Polyp Dataset

Mansoori et al. [125] aimed to reduce reliance on large-scale labeled data and explored the zero-shot learning capability of the Segment Anything Model 2 (SAM 2) in colorectal polyp segmentation. The model achieved excellent segmentation results on six public datasets without additional training, particularly showing a significant improvement in accuracy with bounding box prompts. In the video polyp segmentation task, SAM 2 demonstrated a notable enhancement compared to the original SAM model, with a 31.4% increase in mIoU on the PolypGen dataset. However, the model’s performance is highly dependent on the type of prompts, with significant differences in effectiveness between different prompting methods.

#### 4.5.3. Real-Time Performance of the Model

Banik et al. [126] addressed the issue of deep learning requiring a large number of parameters and training time for polyp segmentation by proposing the Deep Hierarchical Broad Learning System Network. This model enhances segmentation accuracy and efficiency through two multi-scale feature extraction techniques: 2D-DT-CWT and Sifp-conv, and introduces diligent parameter control to stabilize network training. Its flat network structure eliminates the need for multiple layers to learn complex features, resulting in superior performance in terms of computational cost. However, there are still certain limitations in segmentation effectiveness for morphologically complex and relatively flat polyps.

### 4.6. Video Polyp Segmentation

Some researchers have also conducted studies on video polyp segmentation. Ji et al. [22] addressed the issue of the lack of fine-grained segmentation annotations in large-scale datasets by constructing the video polyp segmentation dataset SUN-SEG and proposing the baseline model PNS+. This model utilizes global and local encoders to extract long-term and short-term spatiotemporal information and then progressively refines the segmentation results through a normalized self-attention block. This method achieves a real-time inference speed of 170 fps but lacks robustness when dealing with interference factors such as surgical instruments and optical glare. Wu et al. [127] addressed challenges such as the diversity of polyp types, low contrast, and blurred boundaries while also considering real-time requirements by proposing a lightweight, context-aware network called PolypSeg+. This network employs three key components: Adaptive Scale Context for gathering multi-scale information, Efficient Global Context for reducing background noise and improving feature fusion, and a Feature Pyramid Fusion module that effectively utilizes the extracted contextual features. It achieves high segmentation accuracy while ensuring real-time performance. However, the segmentation results still need improvement when dealing with tiny polyps and extremely low contrast scenarios. The FlowICBNet model was created by Wan et al. [128] to solve the problem of polyp segmentation that is hard to perform in endoscopic videos because the camera shakes and the frames become out of focus. This model uses the Iterative Feedback Unit (IFU) to help with video polyp segmentation and adds Reference Frame Selection (RFS) and Flow-Guided Warping (FGW) modules to use past frame prediction results to help segment the current frame better. This approach demonstrates excellent performance in alleviating the difficulties posed by camera shake and frame defocus. However, further optimization of the IFU’s iterative feedback mechanism is still necessary due to its potential for "over-correction". Lu et al. [129] came up with the Diff-VPS video polyp segmentation network, which is based on a multi-task diffusion model and solves the problems of high camouflage and redundant temporal cues in video polyp segmentation. This method uses a multi-task supervision strategy to add classification and detection tasks to the diffusion model. It also creates a Temporal Reasoning Module (TRM) to obtain more dynamic information from video frame sequences. An adversarial self-supervised strategy improves the accuracy of frame reconstruction, which makes it easier to find polyps that are well hidden. However, only the SUN-SEG dataset has validated this method, necessitating further research to explore its generalization to other datasets and more complex scenarios. Chen et al. [130] addressed the challenge of modeling long-range spatiotemporal relationships in colonoscopy videos by proposing the Mixture-Attention Siamese Transformer (MAST) method. This approach effectively captures and models the long-range spatiotemporal relationships between video frames through a Siamese Transformer architecture and a mixed attention mechanism. It achieved excellent segmentation results on the SUN-SEG dataset. However, due to its validation on a single dataset, its generalization capability still requires further evaluation. To fix the problem that traditional polyp segmentation algorithms do not work as well when switching from static images to video scenes, Xu et al. [131] came up with SSTFB, an end-to-end video polyp segmentation scheme that combines self-supervised learning with spatiotemporal self-attention. This method innovatively employs self-supervised learning as an auxiliary task, combining it with a spatiotemporal self-attention mechanism and feature branching techniques to enhance feature representation capabilities through a joint loss optimization strategy. The method achieved significant improvements on the SUN-SEG dataset and realized real-time inference at 126 frames per second. However, the model may still experience some degree of mis-segmentation under extreme lighting conditions, severe artifacts, and complex background environments. Hu et al. [132] proposed the SALI network to address the issue of limited segmentation accuracy in endoscopic videos caused by significant changes between adjacent frames and consecutive low-quality frames. This method utilizes a short-term alignment module to align features of adjacent frames through deformable convolution and designs a long-term interaction module that combines a memory bank and mask attention to reconstruct more reliable polyp features. This design effectively enhances the stability of spatiotemporal features and improves the handling of significant changes and low-quality frames. However, memory cost constraints limit the memory length to 35 frames, leading to inaccurate segmentation in scenarios with extremely low quality or drastic changes. Yang et al. [133] addressed the challenge of modeling long-term spatiotemporal dependencies in medical video segmentation by proposing the Vivim framework based on Video Vision Mamba. This method captures and compresses long-range spatial and temporal dependencies in videos through the Temporal Mamba Block and ST-Mamba module and introduces boundary-aware affine constraints to reduce prediction uncertainty in ambiguous lesion areas. Vivim achieves efficient long-sequence modeling while maintaining linear computational complexity. However, the overall complexity of the model is relatively high, necessitating a balance between operational efficiency and accuracy.

### 4.7. Remarks

This section summarizes the model methods outlined in this review. Table 2 is a summary of polyp segmentation methods from 2022 to 2024; Table 3 summarizes polyp segmentation methods from 2018 to 2021, and Table 4 summarizes video polyp segmentation methods from 2022 to 2024. In this section, we will summarize the contents of these three tables, discussing their common advantages, common problems, and potential solutions. We will briefly outline the issues and solutions in this section; for a detailed discussion on the ongoing challenges and future trends, please refer to Section 8 and Section 9.

Table 2 summarizes the research progress on polyp segmentation methods from 2022 to 2024, primarily including CNN, Transformer, hybrid architectures, Mamba-based methods, and five other categories. Among these, CNN-based methods mainly focus on feature extraction and optimizing boundary details, but they still have limitations in complex scenarios; Transformer-based methods excel at capturing long-range dependencies but come with high computational costs and perform poorly on small target segmentation; hybrid architectures attempt to combine the local feature extraction capabilities of CNNs with the global modeling capabilities of Transformer to achieve a balance between accuracy and efficiency; meanwhile, emerging Mamba-based methods show potential in long sequence modeling but still require further validation of their robustness. Most of these methods are based on fully supervised strategies and utilize various backbone networks (such as Res2Net, PVTv2, Swin Transformer, etc.). Some works emphasize efficient training in semi-supervised or weakly supervised environments while also exploring scenarios like cross-domain adaptation, real-time segmentation, and few-shot learning. Multi-scale feature fusion and attention mechanisms are widely used to improve the precise localization of morphologically diverse, small-sized, or low-contrast polyp regions. Most methods have been validated on the CVC-ClinicDB and Kvasir-SEG datasets, achieving favorable results. The common advantages of these methods include the following: effective handling of multi-scale features, strong feature extraction and representation capabilities; improved segmentation accuracy through various feature fusion strategies; and some methods achieving good real-time performance. However, these methods also face several common challenges: suboptimal segmentation performance for small polyps; decreased performance in complex scenarios with low contrast and blurred boundaries; inadequate results for simultaneous segmentation of multiple polyps; the need to balance computational efficiency and real-time performance; and limited generalization performance on out-of-domain datasets. Possible solutions to these issues include first developing more effective multi-scale feature extraction and fusion strategies, with a particular focus on feature representation for small targets. Second, introducing stronger boundary-aware mechanisms and contrastive learning strategies. Additionally, designing lightweight yet efficient network structures to balance performance and computational costs. Finally, exploring semi-supervised and weakly supervised methods to reduce annotation dependence and enhancing the model’s domain adaptation capabilities to improve robustness in different scenarios. We anticipate significant advancements in polyp segmentation technology in the future due to these efforts.

Table 3 summarizes various polyp segmentation methods from 2018 to 2021, including TransFuse, PraNet, ResUNet++, and UNet++. These methods are primarily based on convolutional neural network architectures, such as ResNet and Res2Net, and employ fully supervised learning approaches. Each method presents innovative solutions to specific challenges in polyp segmentation: TransFuse integrates multi-layer features through parallel branches and a BiFusion module; PraNet introduces partial decoders and a reverse attention module; ResUNet++ enhances segmentation of polyps of different shapes and sizes using ASPP; and UNet++ redesigns the skip connection paths. A common advantage of these methods is their ability to effectively capture multi-scale features of polyps, improving segmentation accuracy and addressing issues such as diverse polyp shapes and unclear edges to some extent. However, these methods also face shared challenges, including high computational complexity, poor segmentation performance for very tiny targets, and inaccuracies in segmenting complex boundaries. Some ways to solve these problems are to make network architectures that are lighter, come up with better ways to extract and combine features, try out self-supervised or semi-supervised learning methods, use attention mechanisms to improve how features are represented, and make training datasets more diverse and larger. Additionally, integrating the latest deep learning technologies, such as state space models, is expected to further enhance the performance and generalization ability of polyp segmentation. Future research could focus more on model lightweighting, real-time performance, and robustness to extreme conditions, driving the advancement of medical image segmentation technology through interdisciplinary innovative approaches.

Table 4 summarizes various deep learning methods used for video polyp segmentation from 2022 to 2024. These methods include CNN-based PNS+, Mamba-based Vivim, hybrid architecture MAST, FlowICBNet, and SALI, among others. These models primarily utilize backbone networks such as Res2Net and PVTv2, employing either fully supervised or self-supervised learning approaches. By introducing innovative technologies such as spatiotemporal self-attention mechanisms, the Temporal Mamba Block, the Siamese Transformer, and hybrid attention modules, these methods have made significant progress in improving segmentation accuracy and processing efficiency. A common advantage of these methods is their ability to effectively capture the spatiotemporal relationships between video frames, enhancing the accuracy of polyp feature extraction and addressing issues such as the lack of large-scale datasets, low-quality frames, and spatial variations to some extent. However, these methods face common challenges, including high computational complexity, poor segmentation performance for small and highly concealed polyps, and limited model generalization capabilities. Possible solutions to these issues may include introducing more advanced attention mechanisms, such as multi-scale and cross-frame attention; exploring lighter and more efficient network architectures, such as Mamba state space models; developing more robust data augmentation strategies; leveraging self-supervised learning and contrastive learning techniques to enhance the model’s feature representation capabilities; constructing larger and more diverse datasets; and exploring multimodal and cross-modal learning methods. Through continuous innovation and research, it is believed that these challenges can be gradually addressed, leading to more precise video polyp segmentation technology.

## 5. Polyp Segmentation Loss Function

In polyp segmentation, class imbalance is a pervasive challenge: polyp regions typically occupy only approximately 5–15% of the image, leaving background pixels overwhelmingly dominant. This skew biases models toward the background class and diminishes sensitivity to small lesions. Consequently, careful selection of the loss function is critical to mitigate imbalance-induced bias and improve overall detection and segmentation performance.

The main loss functions are as follows:

1. Weighted Binary Cross-Entropy Loss;

(1) $$L_{BCE}=-\alpha \times y\times \log\left(p\right)-(1-\alpha )\times (1-y)\times log(1-p)$$

Here, *y* denotes the ground truth label, *p* represents the probability predicted by the model, and α is the weighting factor for the foreground class (polyp), which is typically set between 0.6 and 0.8.

2. Dice Loss;

Dice loss directly optimizes the overlap between the predicted and ground-truth masks. By emphasizing relative overlap rather than absolute pixel counts, it is inherently more robust to class imbalance.

(2) $$L_{Dice}=1-\frac{2\sum_{i=1}^{N}P_{i}G_{i}}{\sum_{i=1}^{N}P_{i}+\sum_{i=1}^{N}G_{i}}$$

*P_i_* is the probability that the *i*-th pixel belongs to the segmentation region, and *G_i_* is the ground truth value of the *i*-th pixel.

3. Focal Loss;

(3) $$L_{Focal}=-\alpha \times {\left(1-p\right)}^{\gamma }\times y\times \log\left(p\right)-(1-\alpha )\times P^{\gamma }\times (1-y)\times log(1-p)$$

Here, *y* represents the true label, *P* denotes the predicted probability, and α is the class balancing factor, which is typically set to 0.25. By using the modulating factor (1 − *p*)^γ^, the weights of easily classified samples are automatically reduced, allowing the model to focus more on hard-to-classify samples. The parameter *γ* controls the extent of this weighting adjustment between easy and hard samples (usually *γ* = 2), resulting in excellent performance in challenging regions such as polyp boundaries.

4. Tversky Loss;

(4) $$L_{Tversky}=1-\frac{TP}{TP+\alpha \times FP+\beta \times FN}$$

*TP* denotes true positives, *FP* denotes false positives, and *FN* denotes false negatives.

As a generalized form of the Dice loss, the balance between false positives and false negatives can be controlled by adjusting the *α* and *β* parameters. When a higher recall is required (for example, to avoid missing small polyps), set *β > α*.

Single loss functions often struggle to optimize all metrics simultaneously, making combined loss functions a common approach.

(5) $$L_{combined}=\lambda_{1}\times L_{BCE}+\lambda_{2}\times L_{Dice}+\lambda_{3}\times L_{Focal}$$

Here, *λ*_1_, *λ*_2_, and *λ*_3_ represent the combination weights for each loss function, with *λ*_1_ + *λ*_2_ + *λ*_3_ = 1. *L_BCE_*, *L_Dice_*, and *L_Focal_* denote the respective calculated loss values.

## 6. Polyp Segmentation Datasets

**Figure 7:**
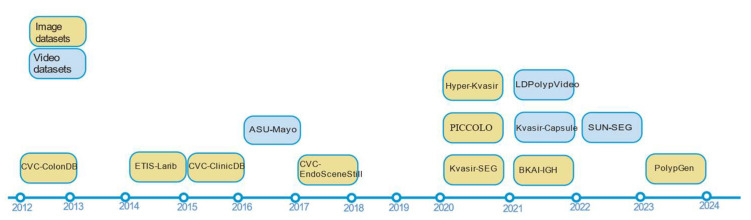
Progress in polyp segmentation datasets.

There are five commonly used image datasets for polyp segmentation, namely CVC-ColonDB [44], ETIS-Larib [139], CVC-ClinicDB [140], CVC-EndoSceneStill [141], and Kvasir-SEG [142]. Some images from CVC-ClinicDB [140] and Kvasir-SEG [142] are commonly used as training data. Figure 7 displays the progress of the polyp segmentation dataset. Table 5 provides detailed information on the related polyp dataset.

### 6.1. Datasets of Polyp Image

The CVC-Colon DB [44] was created by Bernal et al. in 2012 at the Computer Vision Center and Computer Science Department of Universitat Autònoma de Barcelona situated in Barcelona, Spain. The dataset includes 300 images and their corresponding polyp masks were obtained from 13 polyp video sequences acquired from 13 patients. The ETIS-Larib database [139] was developed in 2014 at the ETIS Laboratory of CY Cergy Paris University. This dataset includes 196 annotated samples extracted from 34 colonoscopy videos. The CVC-ClinicDB database [140] was created by Bernal et al. in 2015 in collaboration with a hospital clinic in Barcelona, Spain. The database includes 612 images, which were derived from 31 polyp video sequences collected from 23 patients. CVC-EndoSceneStill [141] is a dataset formed in 2017 by merging CVC-ColonDB and CVC-ClinicDB. It comprises 912 images derived from 44 video sequences obtained from 36 patients. Kvasir-SEG [142] contains 1000 images, annotated by expert scientists from Oslo University Hospital in 2020. The images and videos in Hyper-Kvasir [143] were collected from actual gastroscopy and colonoscopy tests at Norway’s Baerum Hospital from 2008 to 2016 and were partially annotated by experienced gastroenterologists. It includes 11.62 h of video and 1,059,519 video frames. This dataset was published in 2020. The Piccolo dataset [144] contains 3433 clinical colonoscopy video images, including both white light and narrow-band imaging images, which were obtained from colonoscopy procedures performed on human patients. It includes 76 different lesions from 48 patients. This dataset was published in 2020. The BKAI-IGH [145] dataset was published in 2021 and contains 1200 images. The PolypGen dataset [146] was collected from six different centers across Europe and Africa. It contains 1537 polyp images, 2225 positive video sequences, and 4275 negative frames. A total of 3762 positive frames and 4275 negative frames are provided. This dataset was published in 2023.

**Table 5:** Comparative analysis of polyp datasets in existing research studies.

Datasets of Polyp Image/Video	Dataset	Year	Number of Images	Number of Video Sequences	Resolution
Datasets of polyp image	CVC-ColonDB [44]	2012	300	13	574 × 500
	ETIS-Larib [139]	2014	196	34	1225 × 966
	CVC-ClinicDB [140]	2015	612	31	384 × 288
	CVC-EndoSceneStill [141]	2017	912	44	384 × 288 to 574 × 500
	Kvasir-SEG [142]	2020	1000	N/A	332 × 487 to 1920 × 1072
	PICCOLO [144]	2020	3433	40	854 × 480 to 1920 × 1080
	Hyper-Kvasir [143]	2020	110,079	374	332 × 487 to 1920 × 1072
	BKAI-IGH [145]	2021	1200	N/A	1280 × 959
	PolypGen [146]	2023	8037	N/A	384 × 288 to 1920 × 1080
Datasets of polyp video	ASU-Mayo [61]	2016	36,458	38	688 × 550
	LDPolypVideo [147]	2021	40,266	160	560 × 480
	Kvasir-Capsule [148]	2021	4,741,504	117	336×336
	SUN-SEG	2022	158,690	1013	1158 ×1008 to 1240 × 1080

### 6.2. Datasets of Polyp Video

The ASU-Mayo dataset [61] contains 36,458 consecutive frames from 38 videos and provides 3856 labels for 10 frontal videos. This data was published in 2016. LDPolypVideo [147] includes 160 colonoscopy videos and 40,266 frames. This data was published in 2021. Smedsrud et al. in 2021 introduced the Kvasir-Capsule dataset [148], which includes 47,238 labeled frames and 4,694,266 unlabeled frames. It comprises 117 videos, from which a total of 4,741,504 image frames can be extracted. The colonoscopy videos in SUN-SEG [22] come from the Showa University and Nagoya University database (also known as the SUN database [149]). It contains 158,690 colonoscopy video frames. Various types of annotations are provided, including attributes, object masks, boundaries, scribbles, and polygons. This data was published in 2022.

## 7. Polyp Segmentation Evaluation Metrics

The metrics for evaluating polyp segmentation performance include Dice Similarity Coefficient (DSC), Intersection over Union (IoU), Recall (R), Average Precision (AP), Precision (P), Pixel Accuracy, and Frames Per Second (FPS) for assessing real-time performance. Among these, the key metrics are mean Dice and mean IoU.

*Dice* [150,151,152,153] is used for pixel-by-pixel comparison of predicted segmentation results with ground truth data. Its description is as follows:

(6) $$Dice\left(A,B\right)=\frac{2\times |A\cap B|}{\left|A\right|+|B|}=\frac{2\times TP}{2\times TP+FP+FN}$$

where *A* represents the predicted segmentation mask and *B* represents the ground truth segmentation mask. *TP, FP, TN,* and *FN* stand for true positives, false positives, true negatives, and false negatives, respectively. The definitions of *TP, FP, TN*, and *FN* are shown in Table 6.

*IoU* [154] is the number of overlapping pixels between the target mask and the predicted mask divided by the total number of pixels present in both masks. It is represented by the mathematical Formula (7).

(7) $$IoU\left(A,B\right)=\frac{A\cap B}{A\cup B}=\frac{TP}{TP+FP+FN}$$

*Pixel accuracy* [155] is calculated by dividing the number of correct predictions by the total number of predictions. True positives refer to pixels accurately predicted to belong to a specific category, and true negatives refer to pixels identified as not belonging to that category. It is represented by the mathematical Formula (8).

(8) $$Pixel\;accuracy=\frac{TP+TN}{TP+TN+FP+FN}$$

*Precision* is the number of correctly classified positive image samples divided by the number of image samples classified as positive. It is represented by the mathematical Formula (9).

(9) $$Precision=\frac{TP}{TP+FP}$$

*Recall* is the number of image samples correctly classified as positive divided by the total number of positive image samples. It is represented by the mathematical Formula (10).

(10) $$Recall=\frac{TP}{TP+FN}$$

## 8. Polyp Segmentation Model Performance Evaluation

In this section, we selected 44 models from those mentioned in Section 4 to evaluate their segmentation performance and real-time capabilities. Table 7 displays the performance evaluation results of the above 44 models.

From Table 7, DDPM [124] and ISCNet [74] rank top two on the CVC-ClinicDB dataset, with DDPM being 0.6% and 1.7% higher than ISCNet in mDice and mIoU, respectively; Polyper [112], ISCNet [74], and Polyp-Mamba [29] performed outstandingly on the Kvasir-SEG dataset, with Polyper having the highest mDice and mIoU at 0.948 and 0.904; Polyper [112], TGANet [136], and SAM2 showed excellent results on the CVCColonDB dataset, with SAM2 achieving the highest mDice and mIoU at 0.934 and 0.877; Polyper [112] and SAM2 [125] ranked second and first on the ETIS-Larib dataset, with SAM2 being 7.6% and 10.5% higher than Polyper in mDice and mIoU, respectively; Polyper [112], TGANet [136], and Polyp-Mamba [29] performed exceptionally on the EndoScene dataset, with Polyper having the highest mDice at 92.4% and TGANet having the highest mIoU at 89.9%.

We selected ten models from Table 7 and compared performance evaluation metrics on four datasets: CVC-ClinicDB, Kvasir-SEG, CVC-ColonDB, and ETIS-Larib. In comparison to other models, ISCNet and Polyper demonstrate excellent segmentation performance on the CVC-ClinicDB and Kvasir-SEG datasets. On the CVC-ColonDB and ETIS-Larib datasets, SAM2 achieves the best segmentation performance.

**Figure 8:**
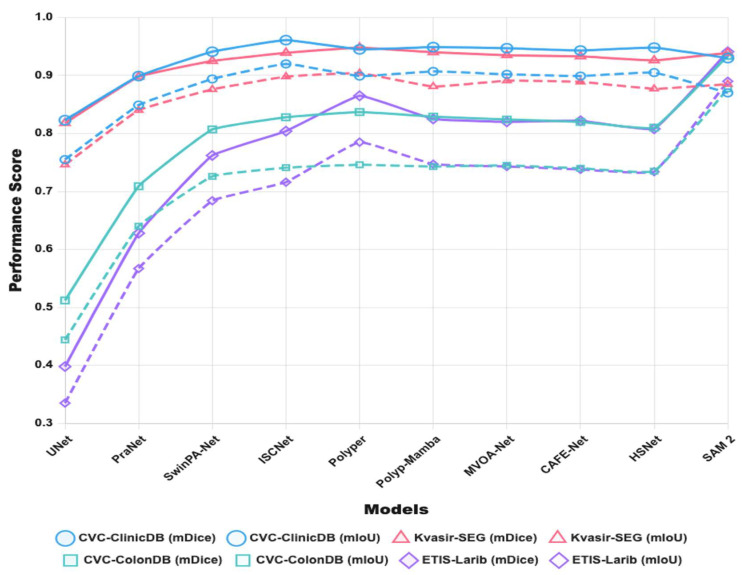
Comparative performance analysis of 10 polyp segmentation models across four benchmark datasets. Each dataset is represented by two metrics: mDice (solid lines) and mIoU (dashed lines).

Figure 8 presents a comparative analysis of the performance of ten polyp segmentation models across four benchmark datasets. On the CVC-ClinicDB dataset, the ISCNet model performed exceptionally well, achieving mDice and mIoU scores of 96.1% and 92%, respectively. However, it was still slightly lower than the DDPM model by 0.6% and 1.7%. On the Kvasir-SEG dataset, Polyper demonstrated the best performance, followed by Polyp-Mamba. SAM2 excelled on the CVC-ColonDB dataset, achieving the highest mDice of 93.4% and the highest mIoU of 87.7%. SAM2 also achieved outstanding performance on the ETIS-Larib dataset, with mDice and mIoU scores of 94.1% and 89%, respectively.

**Figure 9:**
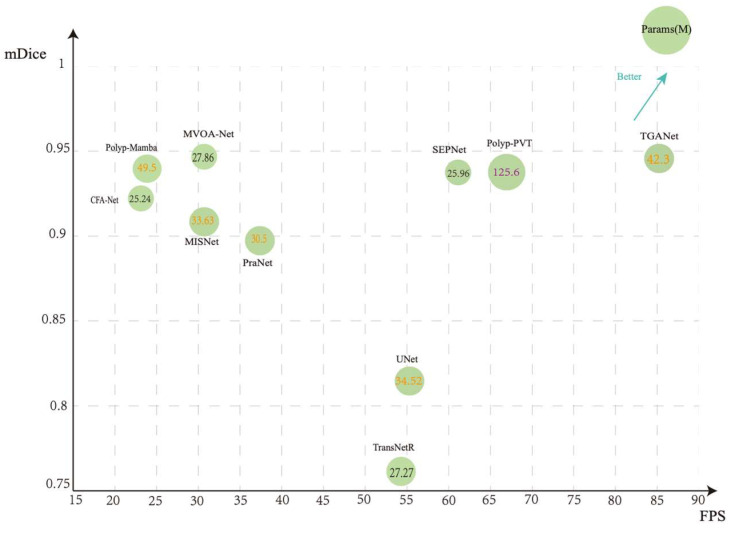
Distribution of Params, FPS, and mDice values for 10 models on the CVC-ClinicDB dataset.

Based on Table 7 and Table 8, we selected ten models to analyze the relationship between the segmentation performance metrics and real-time capabilities. As shown in Figure 9, a higher mDice and mIoU segmentation metric indicates better segmentation performance for the model. For models with comparable segmentation performance, a higher FPS real-time metric, along with a moderate number of model parameters, suggests a greater potential for clinical application of that model.

The size of the parameters is an important indicator of model complexity. As shown in Table 8, the number of parameters in the models ranges from 2.54M to 287.75M. Smaller models, such as PolypSeg+ with 2.54M parameters and ERDUNet with 10.21M parameters, have an advantage in terms of parameter count and may perform better in environments with limited memory and computational resources. Larger models, like HST-MRF with 174.73M parameters and DS-TransUNet with 287.75M parameters, may be better at feature extraction and segmentation accuracy, even though they have more parameters. Therefore, when selecting a model, it is necessary to strike a balance between performance and resource consumption.

Flops is another important metric for evaluating the computational complexity of a model. As shown in Table 8, the range of Flops varies from 7.23G (PolypSeg+) to 55.36G (CFA-Net). A higher Flops value indicates that the model requires more computational resources during inference, which may affect real-time performance. For applications that require quick responses, it may be more appropriate to choose a model with lower Flops.

FPS is a direct indicator of a model’s real-time performance. As shown in Table 8, the FPS ranges from 2.06 (I-RA) to 126.00 (SSTFB). A high FPS value indicates that the model can process more frames in less time, making it suitable for real-time monitoring and diagnostic applications. 

When selecting a polyp segmentation model, it is important to balance parameter size, computational complexity, and real-time performance based on specific application needs. Future research could further explore how to optimize a model’s real-time performance while maintaining high segmentation accuracy to meet the demands of clinical applications.

The distribution in Figure 9 shows the performance of different models used in polyp segmentation, where the mDice value represents the segmentation performance of polyps and FPS indicates the real-time processing capability of the model. Among the 10 models surveyed, TGANet performed excellently, with moderate parameter count, and achieved the highest mDice and FPS values. In clinical applications, the model’s parameter count cannot be too large, and it must maintain a balance between real-time processing speed and segmentation capability.

## 9. Open Research Challenges

Polyp segmentation can assist medical professionals in analyzing and diagnosing polyps. Over the past decade, deep learning-based polyp segmentation technology has rapidly advanced. Although current segmentation models perform well, there is still room for improvement in segmenting extremely challenging images that exhibit blurred boundaries, low contrast, or very small polyp sizes, and there are many challenges to successfully applying this technology in clinical settings.

### 9.1. Lack of Large-Scale Datasets

High-quality polyp segmentation annotation data requires the involvement of specialized doctors. However, the annotation process is often time-consuming, labor-intensive, and costly, which limits the scale and quality of training data for models. Additionally, different doctors may have discrepancies in annotating polyps, leading to inconsistencies in the training data. Currently, existing datasets have insufficient sample sizes and lack diversity, which may not cover various types, shapes, and sizes of polyps. In real clinical environments, doctors may observe multiple types of polyps in the same image. However, many datasets frequently feature images that highlight only a single type of polyp. For example, the current ETIS-LaribPolypDB, CVC-ClinicDB, and Kvasir-SEG datasets primarily consist of images of single polyps. Multiple polyps can appear simultaneously in endoscopic images, exhibiting diverse characteristics in terms of size (e.g., <5 mm vs. 10–15 mm), color (e.g., red, pink, light brown), and shape (e.g., flat, mushroom-like, stalked). Furthermore, an endoscopic image may simultaneously feature adenomatous polyps and hyperplastic polyps. However, the limited number of images depicting multiple polyps in existing datasets hinders models from concurrently learning how to handle different types of polyps during training. Due to the lack of diverse samples, models may struggle to accurately identify and segment different types of polyps in practical applications, thereby affecting the accuracy of clinical decision-making.

### 9.2. Image Quality and Noise

Various factors, particularly changes in lighting, endoscopic angles, and interference from intestinal contents, affect the image quality in the polyp segmentation dataset. During endoscopic examinations, the intensity and angle of the light source may vary. For instance, when the endoscope is close to a polyp, the light may be too intense, causing excessive reflection from the polyp’s surface and creating highlights; conversely, when the endoscope is farther away, insufficient light may lead to an overall dark image with lost details. These lighting variations can cause the boundaries of the polyp to become blurred, making accurate segmentation difficult. Additionally, changes in the endoscopic angle can also impact image clarity. When the endoscope enters the intestine at different angles, it may distort the shape and position of the polyp, especially in curved sections of the intestine. This change in perspective can make the polyp’s morphology irregular, increasing the difficulty of segmentation. Furthermore, liquids, gases, or other contents within the intestine may interfere with the clarity of the endoscopic images. For example, liquid in the intestine can cause light scattering, resulting in a blurred image. These contents may obscure the polyp, preventing segmentation algorithms from accurately identifying the polyp’s boundaries. In existing datasets, some images in the Kvasir-SEG dataset exhibit uneven lighting, certain images in the CVC-ClinicDB dataset show perspective distortion, and the ETIS-LaribPolypDB dataset contains images with occluded contents. For instance, intestinal contents partially obscure a polyp in one endoscopic image, and due to insufficient lighting, the polyp’s color contrast with the surrounding tissue is low, resulting in unclear boundaries. In such cases, the segmentation model may mistakenly classify the polyp as background or fail to accurately identify its shape and size. In summary, these factors collectively contribute to the decline in image quality within the polyp segmentation dataset, which in turn affects the training and testing performance of models. Therefore, improving image acquisition techniques, enhancing image processing algorithms, and constructing higher-quality datasets are key to increasing the accuracy of polyp segmentation.

### 9.3. Complex Shapes and Boundaries

The differences in the shape, size, and color of polyps have a significant impact on the polyp segmentation task. Firstly, variations in the shape of polyps can affect the difficulty of edge detection. For example, flat polyps typically present as flat surfaces with blurred edges, nearly level with the intestinal wall, which may confuse them with surrounding tissues, making it challenging for segmentation algorithms to accurately identify them. In contrast, mushroom-shaped polyps have a distinct stalk and umbrella-like top with clear edges, while stalk-type polyps have a slender stalk and a smaller top, giving them a more unique shape. These shape differences may require different segmentation strategies, thereby increasing the complexity of the model. Secondly, differences in the size of polyps can also affect segmentation outcomes. The surrounding tissues may obscure polyps smaller than 5 mm, making them easily overlooked, whereas polyps measuring 10-15 mm are relatively easier to identify. Polyps larger than 20 mm are usually easier to segment but may overlap with other structures, leading to identification difficulties. Noise or other tissues in images may mask small polyps, causing segmentation algorithms to miss them, while larger polyps may obscure smaller surrounding polyps, thereby affecting the segmentation accuracy. Additionally, differences in the color of polyps can similarly influence segmentation results. Red polyps, due to their rich vascularity and bright color, are simple to identify; however, pink polyps may be difficult to distinguish from surrounding normal tissues due to their similar color. Light brown polyps may appear darker due to tissue degeneration or inflammation. These color differences can lead to insufficient contrast and color distortion, making it hard to differentiate polyps of similar colors from surrounding tissues, thus reducing the accuracy of segmentation algorithms. Variations in lighting or endoscopic reflections may also result in the loss of color information, further impacting segmentation performance. In the same image, there may be polyps of different shapes, sizes, and colors simultaneously, which increases the complexity of the segmentation algorithm and its generalization capability. Segmentation algorithms need to possess multi-scale perception abilities to handle various types of polyps, which adds to the difficulty of model training.

### 9.4. Insufficient Generalization Ability

In the polyp segmentation task, the model’s generalization ability is a key factor. There are significant differences between different datasets; for example, the Kvasir-SEG dataset contains endoscopic images, while the CVC-ClinicDB dataset consists of clinically collected images. The distribution of polyp types and image resolution reflect these differences, resulting in variations in dataset characteristics. For instance, a model trained on the Kvasir-SEG dataset shows a significant drop in segmentation performance when tested on the CVC-ClinicDB dataset, especially in recognizing small or irregularly shaped polyps. Also, models trained on the CVC-ClinicDB dataset tend to focus on certain types of polyps, like mushroom-shaped ones. This means that, when tested on the ETIS-LaribPolypDB dataset, they perform less well, especially when dealing with polyps that are very different in color and shape. Training and testing on different endoscopic devices can also lead to a decrease in segmentation accuracy. Insufficient generalization ability can result in inaccurate edge detection and difficulties in recognizing small polyps. The model may struggle to accurately identify the edges of polyps, leading to blurred segmentation results. In the training dataset, there are fewer samples of small polyps, which further weakens the model’s ability to recognize them. The main reasons for insufficient generalization ability include data distribution shifts and limitations in feature learning. Training a model on a specific dataset may prevent it from learning the features of polyps with different shapes and colors, which could impact its performance in real-world applications.

### 9.5. High Real-Time Requirements

In polyp segmentation models, scenarios with high real-time requirements pose a severe challenge to computational efficiency. During colonoscopy, doctors need to observe and analyze the condition of polyps in real time to make timely decisions. Therefore, the segmentation model must be capable of processing images and providing segmentation results within a few seconds. Additionally, automated endoscopic systems need to analyze video streams in real-time to quickly identify and label polyps, thereby assisting doctors in diagnosis. To improve processing speed, it may be necessary to simplify the model, which could lead to a decrease in segmentation accuracy and an increased risk of misdiagnosis. During endoscopic examinations, images change rapidly, and insufficient real-time processing capability may prevent the model from accurately capturing the dynamic features of polyps. Thus, polyp segmentation models face an important challenge of maintaining segmentation accuracy while ensuring real-time performance.

### 9.6. Lack of Interpretability

Doctors often view deep learning models as “black boxes”, lacking interpretability, making it challenging to comprehend the models’ decision-making process. This “black box” issue limits clinical trust, as the internal decision-making process is opaque, and doctors cannot clearly comprehend how the model arrives at a particular segmentation result. For instance, in an endoscopic image, the model may label certain areas as polyps, while these areas do not align with the characteristics of polyps based on the doctor’s experience. The lack of interpretability leads to a decrease in doctors’ trust in the model, creating barriers to clinical application. During critical decision-making, doctors may become skeptical of the model’s judgments and thus be reluctant to rely on its results, which will affect the model’s practical application. If doctors do not trust the model, it may lead to the oversight of potential polyps, ultimately impacting patient health. Therefore, the interpretability of polyp segmentation models is crucial for clinical application. Enhancing the model’s transparency and interpretability can strengthen doctors’ trust, thereby improving the model’s effectiveness in real medical settings. Therefore, the interpretability of polyp segmentation models is crucial for clinical application. Recent research has made significant progress in addressing this challenge. Wickstrøm et al. [156] developed methods combining uncertainty estimation with interpretability techniques for colorectal polyp segmentation, using Monte Carlo Dropout for uncertainty quantification and Guided Backpropagation to visualize which input features the model deems important for predictions. Their findings revealed that models primarily utilize shape and edge information of polyps, and importantly, erroneous predictions exhibit significantly higher uncertainty levels, providing clinicians with valuable confidence indicators. Furthermore, Ramos and Hortúa [157] advanced this field by implementing Bayesian neural networks with multiplicative normalized flows, achieving well-calibrated uncertainty estimates while maintaining high segmentation performance, demonstrating that interpretability and uncertainty quantification can be effectively integrated without compromising accuracy. Enhancing the model’s transparency and interpretability can strengthen doctors’ trust, thereby improving the model’s effectiveness in real medical settings.

### 9.7. Ethics and Privacy

Due to ethical and privacy concerns, the use of medical data is subject to strict limitations [158,159,160]. Sensitive personal information often resides in medical data, necessitating protection and preventing its use for research or commercial purposes without patient consent [161,162]. Therefore, medical research typically requires review by an ethics committee to ensure that the design and implementation of the study do not harm patients or infringe on their rights [163]. This process can lead to delays and complexities in data acquisition. Ethical and privacy issues directly impact the training and performance of deep learning models for polyp segmentation. Researchers may not be able to obtain sufficient labeled data to train deep learning models [164]; a lack of data can prevent the model from learning enough features, thereby affecting its performance. With limited training data, the model may overfit to the training set, resulting in poor generalization to new data [165]. This means that the model may not effectively identify and segment polyps in practical applications. Additionally, if the available dataset lacks diversity, the model may struggle to adapt to various clinical scenarios, reducing its effectiveness in real-world applications [166].

## 10. Future Research Directions

Based on the challenges mentioned in the previous chapter, we explore potential research directions for addressing these challenges in this section.

### 10.1. Develop Larger-Scale and More Diverse Datasets

During the annotation process of the polyp dataset, a multi-expert annotation mechanism was introduced, along with the use of ensemble learning methods and the exploration of automated annotation techniques, aiming to improve the quality of the dataset annotations and address the potential discrepancies that may arise from different doctors annotating polyp images. Variations in doctors’ experiences, knowledge backgrounds, or personal judgments can lead to inconsistent annotation results for the same polyp. By employing multi-expert annotations, collective intelligence can be harnessed to reduce this subjectivity. Additionally, using statistical methods to assess the consistency of annotations among different experts helps identify and analyze the reasons for annotation discrepancies. Experts can combine automated annotation techniques like deep learning with manual review to further enhance annotation quality. Automated annotation systems can continuously learn and optimize based on expert feedback, gradually reducing annotation differences. This comprehensive approach not only improves the accuracy and consistency of annotations but also enhances the quality of the dataset, providing a more reliable foundation for subsequent deep learning model training.

To address the issues of insufficient sample size and lack of diversity in existing datasets, it is particularly important to establish a larger-scale dataset that includes diverse polyp images. On one hand, for the acquired blurry images or blurred video frames, data augmentation methods can be employed to improve image quality. For example, image enhancement techniques can be applied to unclear polyp images to enhance image quality and optimize polyp feature extraction, improving the distinction between polyps and the background, and highlighting the edges and texture features of the polyps. The EndoUIC method proposed by Bai et al. [167] aims to achieve better imaging results; Shang et al. [168] proposed a video deblurring method to recover continuous clear frames from blurry videos; ELKarazle et al. [169] addressed the issue of specular reflection areas in colonoscopy images to improve the readability of medical images; Sharma et al. [170] improved polyp synthesis by controlling the size, shape, and position of polyps. On the other hand, it is also crucial to develop and expand the scale and diversity of the dataset, including images of various polyp categories. For instance, the ColonINST [171] dataset proposed by Ji et al. is a multi-modal instruction-tuning dataset that contains 62 categories and 303,001 colonoscopy images, which can be used for segmentation tasks. These efforts will contribute to enhancing the performance and applicability of polyp segmentation models.

### 10.2. Exploring and Improving Deep Learning Architectures

Refining deep learning architectures and incorporating attention mechanisms can enhance the performance of polyp segmentation models in addressing issues such as small-sized polyps, low contrast, and blurred boundaries. For example, using frequency feature attention modules [91] and parallel attention mechanisms [80] can help the model focus on important features, especially when it comes to small polyps. Additionally, utilizing multi-scale feature extraction methods through a local and global dual-branch structure can efficiently capture features at different scales, combined with multi-scale fusion modules to capture the details of small-sized polyps, thereby enhancing the model’s ability to recognize polyps of varying sizes.

Integrating contextual information is also an effective strategy for improving model performance. For example, [75] use a global guided context aggregation module that can help the model understand the relationship between polyps and their surrounding background, thus improving segmentation results for low-contrast images. To address color variations, specialized modules can be designed, such as dataset-level color augmentation [70], to enhance the color diversity of training data, thereby improving feature extraction capabilities for low-contrast images. Furthermore, designing shape feature extraction modules, such as [121] using DCT for multi-spectral analysis, can enhance the boundaries of blurred areas.

In terms of boundary handling, a variable Laplacian feature refinement module [66] can capture high-frequency information of boundaries, aiding the model in better recognizing the geometric features of polyps, especially when dealing with blurred boundaries. By optimizing segmentation strategies, the accuracy and robustness of segmentation can be further improved. For instance, considering ensemble learning methods that combine predictions from multiple models through voting or weighted averaging, as demonstrated in a strategy involving multiple resampling and majority voting [124], can generate more precise segmentation results. Additionally, selecting or designing appropriate loss functions, such as joint loss optimization strategies [131], can enhance feature representation, thereby improving segmentation accuracy for small-sized polyps.

Prompt-based architectures, such as fine-tuning SAM [172], may also prove to be an effective strategy. Rahman et al. [97] proposed a robust fine-tuning technique for segmentation tasks involving multiple polyps. Future research could develop a self-prompting SAM to automatically generate high-quality prompts, thereby further enhancing segmentation quality. The comprehensive application of these methods will contribute to improving the overall performance of polyp segmentation models.

### 10.3. Weak Supervised

Through weakly supervised and unsupervised learning, polyp segmentation models can be effectively trained using only a small amount of labeled data. Traditional, fully supervised learning methods require a large number of labeled images for training, and most current models still rely on comprehensive supervised learning. However, due to the high cost of labeled data, weakly supervised and semi-supervised learning techniques provide an effective alternative. These techniques can utilize a small amount of labeled data along with a large amount of unlabeled data for model training and generate pseudo-labels for the unlabeled data. These pseudo-labels can serve as additional training data, helping the model learn more features and thereby improving segmentation performance. For example, Hu et al. [173] introduced monotonicity constraints into weakly supervised segmentation, proposing a new method for handling imprecise annotations. It is anticipated that there will be more innovative research in the fields of weakly supervised, unsupervised learning, and hybrid supervision strategies. This research will provide new ideas and methods for training polyp segmentation models, further advancing the development of this field.

### 10.4. Combine with Prior Knowledge

Combining polyp segmentation models with doctors’ prior knowledge—specifically incorporating their expertise and experience during the model’s training and inference processes—can effectively utilize the morphological features and positional information of polyps to guide segmentation. This approach not only enhances the model’s focus on boundary region features but also improves segmentation accuracy and robustness. For instance, doctors often leverage the inherent characteristics of the internal regions of polyps in clinical practice to address boundary ambiguity. To this end, Polyper [112] utilizes features from the internal polyp regions to enhance boundary recognition, resulting in favorable segmentation outcomes. Additionally, bounding box annotations provide relatively accurate semantic and positional information, serving as valuable prior knowledge. W-PolypBox [174] employs the prior information from bounding boxes to enhance the training process for polyp segmentation. This method fully leverages doctors’ expertise, making the model more effective in handling complex medical images, thereby improving polyp segmentation results. By integrating doctors’ experience with deep learning models, the performance of polyp segmentation can be further enhanced, providing more reliable support for clinical applications.

### 10.5. Domain Adaptation

Domain adaptation is a transfer learning technique aimed at improving model performance in target domains, especially when labeled data in the target domain is scarce. 

This method enables the training of models in a source domain, typically rich in labeled data, and then transfers their knowledge to the target domain. The advantages of domain adaptation include reducing the need for labeled data in the target domain, enhancing the model’s generalization ability, and increasing the model’s robustness, all of which can significantly improve the performance of polyp segmentation models. Researchers have proposed various domain adaptation methods [87,175,176,177,178] to address the performance decline caused by domain bias. For example, Pan et al. [179] utilized a frequency-based Federated Domain Generalization framework to tackle the domain generalization problem, while Li et al. [180] compensated for domain gaps through continual learning and multi-scale domain adaptation. By aligning the features of the source and target domains and reducing the distribution differences between them, the model’s performance in the target domain is enhanced, thereby improving the accuracy and reliability of polyp segmentation. These studies indicate that domain adaptation techniques hold significant potential for enhancing applications in medical image analysis, particularly in data-scarce situations.

### 10.6. Develop Lightweight Models

To improve the real-time performance of polyp segmentation, developing lightweight models is particularly necessary. In clinical applications, doctors need to quickly obtain polyp segmentation results to make timely diagnostic and treatment decisions. Lightweight models can process images in a shorter time, thus meeting the requirements for real-time performance. To achieve this, methods such as model compression, network architecture optimization, feature selection, and the use of lightweight convolutions can be considered to design efficient lightweight models. For example, Polyp-LVT [3] replaces the attention layer with a global max pooling layer in the encoder, significantly reducing the model’s computational complexity and parameter count. This type of lightweight model not only improves processing speed but also maintains high segmentation accuracy, thereby providing a better user experience and decision support in clinical applications. These improvements enhance the application potential of lightweight models in real medical environments.

### 10.7. Interpretability

The interpretability of polyp segmentation models refers to the ability of the model to clearly explain its decision-making process and output results during polyp segmentation. This interpretability is particularly important in medical image analysis, as doctors and clinical experts need to understand the basis of the model’s judgments in order to trust its results and make corresponding clinical decisions. To enhance the interpretability of the model, various methods can be employed. First, visualization techniques, such as heatmaps, can be used to show the areas of the image that the model focuses on when making segmentation decisions.

Additionally, using model architectures that are inherently more interpretable for preliminary analysis can help in understanding the decision-making process of the model. Recent advances have shown promising directions in this area. Wickstrøm et al. [156] proposed Monte Carlo Guided Backpropagation, a novel method that not only identifies important input features but also quantifies the uncertainty associated with these feature importances, enabling clinicians to understand both what the model is looking at and how confident it is about these features. This dual insight is particularly valuable when models encounter challenging cases such as small or poorly visible polyps. Moreover, Ramos and Hortúa [157] demonstrated that Bayesian approaches using multiplicative normalized flows can provide well-calibrated predictions with interpretable uncertainty maps, showing higher uncertainty at polyp boundaries and in difficult-to-detect regions, which aligns with clinical intuition and enhances trust in model decisions. Incorporating clinical explanations into the model’s outputs, which align with the clinical experience of doctors, further enhances interpretability. At the same time, continuous evaluation and improvement of the model’s interpretability through feedback from doctors is essential to ensure its effectiveness and reliability in practical applications. In [181], it has been summarized that employing uncertainty quantification methods to assess the reliability of machine learning models can improve the interpretability and acceptability of model results. In summary, these methods can effectively enhance the interpretability of polyp segmentation models, thereby promoting their application in clinical practice.

### 10.8. Federated Learning

Federated learning [182,183,184] is a distributed machine learning method designed to enhance model performance while protecting user privacy. This approach allows multiple devices, such as hospitals and medical institutions, to train models locally without the need to centralize data on a single server. Each device uses local data for training and sends the resulting model updates to a central server, which aggregates these updates to form a global model. In federated learning, the transmission of model updates employs encryption techniques, such as homomorphic encryption or differential privacy, to protect the content of the updates, ensuring that users’ original data remains on local devices, thereby mitigating the risk of data leakage. Furthermore, federated learning can collect data from various medical institutions, where patient populations in different hospitals may exhibit different types and characteristics of polyps. This diversity allows the model to learn more comprehensive features, thereby enhancing its generalization ability. Stelter et al. [185] analyzed the impact of federated learning on polyp segmentation, noting that this method effectively protects patient data privacy. However, the study also mentioned that the controlled noise introduced by differential privacy during training might reduce the model’s accuracy. To address this, the research suggests that future work could utilize Generative Adversarial Networks to generate privacy-preserving data. By increasing the training data for the model while safeguarding data privacy, the segmentation performance and generalization ability of the polyp segmentation model can be further improved.

Polyp segmentation remains a challenging research area that requires the integration of various technical approaches to achieve breakthroughs. Future research trends will place greater emphasis on the robustness, real-time performance, and interpretability of models to promote the widespread application of polyp segmentation technology in clinical settings.

## 11. Conclusions

This article reviews polyp segmentation models based on deep learning methods and the Mamba method, providing a comprehensive overview of research published between 2018 and 2024. First, the research on polyp segmentation models is categorized from the perspective of network architecture and the problems addressed by the models. Second, a comparative analysis of different polyp segmentation models is conducted, with performance evaluation including segmentation performance metrics, model parameters, and real-time capabilities. Third, we summarize commonly used datasets in polyp segmentation for researchers’ reference. Finally, we discuss the main challenges faced in the field of polyp segmentation and future directions for addressing these challenges.

## Figures and Tables

**Table 1:** Summary table of previous reviews on polyp segmentation.

Title of the Paper	Year	Description	Limitations
Deep learning to find colorectal polyps in colonoscopy: A systematic literature review [30]	2020	This review analyzes 35 papers published between 2015 and 2018 that include polyp detection, localization, and segmentation.	The number of analytical studies is relatively small; these are models from 2015–2018.
A survey on deep learning for polyp segmentation: Techniques, challenges, and future trends [31]	2024	This review examines 58 papers from 2019 to 2023 and analyzes 24 models.	There has been no evaluation or analysis of the model’s real-time performance.
Colorectal Polyp Segmentation in the Deep Learning Era: A Comprehensive Survey [32]	2024	This review examines 115 papers from 2014 to 2023 and analyzes 40 model papers from 2015 to 2023.	The models being analyzed do not have real-time performance evaluation.
A systematic review of deep learning based image segmentation to detect polyp [33]	2024	This review examines 117 papers from 2018 to 2023 on the use of deep learning image segmentation models for polyp segmentation. It analyzes 16 models.	The number of models in the statistical table is relatively small.
A Comprehensive Review of Deep Learning for Medical Image Segmentation [34]	2024	This review analyzes 41 medical image segmentation papers from 2015 to 2024, covering skin lesions, hippocampus, tumors, and polyps.	The polyp segmentation model has a small sample size; there is no performance evaluation analysis.
A survey of deep learning algorithms for colorectal polyp segmentation [35]	2024	This review discusses four challenges encountered by deep learning methods in the task of colorectal polyp segmentation, along with related papers.	There is no statistical examination of Mamba-based models, no model statistical table, and no performance evaluation analysis of the model.
Cross-modal hybrid architectures for gastrointestinal tract image analysis: A systematic review and futuristic applications [36]	2024	This review surveys CNN and Transformer methods for organ and polyp segmentation.	There is no statistical examination of Mamba-based models and no assessment of the models’ performance.

**Table 2:** Summary of polyp segmentation methods from 2022 to 2024.

Architecture	Year	Methods	Backbone	Problems Solved	Advantages	Limitations
CNN	2024	RGIA [86]	N/A	Domain transfer problem.	Accurately segmenting polyps under complex shapes and various lighting conditions.	Insufficient handling of domain shift factors such as color and shape differences.
		PolypMixNet [85]	ResNet34	Limited annotated data and class imbalance.	Achieving near fully supervised model performance with just 15% labeled data.	Performance still lags behind fully supervised methods on some datasets.
		DEC-Seg [80]	Res2Net	Changes in the shape, size, and location of polyps; pixel-by-pixel annotation.	Significantly improves segmentation performance with 10% and 30% labeled data.	Challenges remain in handling fuzzy areas and unclear boundaries.
		MEGANet [63]	ResNet-34 and Res2Net-50	Complex background, varying sizes and shapes of polyps, unclear boundaries.	Edge-guided attention; Laplacian operator to enhance feature extraction of polyp boundaries.	Insufficient real-time performance.
		NPD-Net [82]	Res2Net50	Overfitting.	Unsupervised deep supervision strategy.	The segmentation ability will decrease on abnormal images.
		DCL-PS [87]	ResNet-101	Distribution differences between the source domain and the target domain.	Better cross-domain feature alignment.	The edge details of large polyps are not captured accurately enough.
		DLCA_ CMAM [69]	ConvNeXt	The color appearance in the polyp dataset is imbalanced.	Color statistics knowledge for data augmentation.	DLCA requires more computation time compared to Color Jittering.
		MISNet [64]	Res2Net	The location, size, and shape of the polyps vary; the boundaries are unclear.	Adaptive fusion of multi-layer features; accurately capturing the details of polyp boundaries.	Segmentation performance is poor in extremely low-contrast environments.
		WDFF-Net [24]	HarDNet68	Polyps exhibit significant differences in color, size, shape, appearance, and location.	The dual-branch architecture (PFF and SFF) has strong complementarity.	The FPS performance is slightly inferior to that of lightweight models like HarDNet-MSEG.
		UHA-Net [70]	Res2Net-50	Scale variation of lesion areas.	The segmentation effect of small polyps is good.	Complete pixel-level annotated data is needed for training.
		SEPNet [71]	PVTv2-B2	Difficult to learn accurate semantics.	Handling polyps with similar backgrounds.	Loss of edge details.
		FoBS [65]	DeepLabV3+	The appearance of polyps varies.	Multilevel boundary enhancement.	Noise-sensitive.
	2023	S^2^ME [84]	ResNet50	Existing weak supervision methods are mainly limited to spatial domain learning.	Spatial-frequency dual branch; pixel-level adaptive fusion strategy.	Lack of comparison with the latest methods.
		EMTSNet [134]	Res2Net50	How to accurately segment polyps.	Effective extraction of multi-scale features.	The segmentation results for small-sized polyps are not satisfactory.
		GAN-PSNet [135]	Res2Net50	Polyps with variable appearance.	Attention mechanism integrated into the discriminator.	Higher time complexity.
		FEGNet [72]	Res2Net50	The shape, size, and texture of polyps vary; the boundaries are unclear.	Skilled in handling the segmentation of complex-shaped and small-sized polyps.	Insufficient generalization performance.
		CoInNet [73]	DenseNet121	The shape, color, size, and texture of polyps vary, and their boundaries are unclear.	It has excellent segmentation capability for small polyps with an area of ≤1000 pixels.	Detection difficulties in specific scenarios.
		RPANet [88]	ResNet101	Domain adaptation for polyp segmentation models.	No source domain data is required, suitable for cross-hospital deployment.	Insufficient handling of situations where the foreground and background have minimal differences.
		CFA-Net [66]	Res2Net-50	Polyps vary in shape and size, with indistinct boundaries.	Effective in handling small polyps and unclear boundaries.	The segmentation effect of large polyps is not ideal.
		PolypSeg+ [127]	ResNet50	Polyps are diverse, with low contrast against the background and blurred boundaries.	High segmentation accuracy; good real-time performance.	The segmentation effect of very small polyps is poor.
		FTMF-Net [79]	Res2Net	Boundary details; global context information.	Boundary feature extraction from frequency domain perspective.	The segmentation of complex samples still needs improvement.
		ISCNet [74]	ResNet34	Low contrast, small size, and a wide variety.	Performs well when treating small polyps.	Segmentation errors still exist in the edge regions.
		I-RA [77]	Res2Net50	Representation of directionality, singularity, and regularity in the spectral domain.	Contour knowledge guidance enhances spectral domain feature representation.	Real-time performance still needs improvement.
		WS- DefSegNet [81]	Res2net	Pixel-wise annotated dataset.	Weakly supervised and semi-supervised polyp segmentation framework.	The performance on certain datasets is still lower than that of state-of-the-art fully supervised methods.
		ERDUnet [23]	Unet	Extracting global contextual features is difficult; the parameters are too large to be applied clinically.	The model has a small parameter size and achieves an average FPS of 40.71, accurately segmenting large-scale targets.	Recognition errors may occur when the target and background features are too similar.
	2022	LDNet [75]	Res2Net50	The diversity of polyps in shape, size, and brightness, as well as their subtle contrast with the background.	Dynamic kernel generation and update mechanism; efficient self-attention and lesion-aware cross-attention.	The real-time performance and computational efficiency of the model have not been discussed in detail.
		MSRF-Net [76]	N/A	Targets of different sizes; difficult to train on small-scale and biased datasets.	The dual-scale dense fusion; the shape stream.	The performance of low-contrast image segmentation is weak.
		BSCA-Net [68]	Res2Net50	Extraction of boundary information.	Obtain geometric information from multiple angles.	Loss of some low-level details
		AMNet [78]	Res2Net50	Small polyps; similar to the surrounding environment.	Multi-scale fusion; parallel attention mechanism; reverse context fusion.	Performance still needs to be improved in complex real-world environments.
		BCNet [67]	Res2Net50	Inaccurate segmentation.	Excels at handling ambiguous boundaries and complex backgrounds.	There are still cases of inaccurate segmentation in specific complex scenarios.
		NIP [83]	ResNet101	Limited training data, significant variation in polyps, and class imbalance.	MLMix data augmentation methods and CAR resampling strategies.	Real-time performance has not been evaluated in detail.
		TGANet [136]	ResNet50	Changes in polyp size can affect model training.	Outstanding performance in the segmentation of small and flat polyps.	Real-time performance metrics not discussed in detail.
Transformer	2024	CTNet [28]	MiT-b3	Lack of features with advanced semantic details.	Supervised contrastive learning strategy.	Small polyp segmentation is weak.
		HST-MRF [99]	Swin Transformer	Feature information loss problem caused by patch segmentation.	Multi-receptive field patch segmentation strategy.	Relatively weak in precision metrics.
		MLFF-Net [90]	PVTv2	Insufficient feature utilization and feature fusion conflicts.	Multi-layer feature fusion and multi-scale attention.	The segmentation effect of multiple polyps is not ideal.
		PSTNet [91]	Shunted Transformer	Feature misalignment in multi-scale aggregation.	Enhance noise suppression capabilities.	Need to improve computational efficiency.
		Polyp-LVT [3]	PVTv2	Application limitations in clinical settings.	The parameter count is reduced by approximately 44% compared to the baseline model Polyp-PVT.	There is still room for improvement in accuracy.
		PFENet [92]	PVTv2-B2	Limitations of global context modeling and cross-layer feature interactions.	The CFE module enhances feature representation capabilities; the CMG module fuses features.	Decline in out-of-domain generalization performance.
		VANet [96]	CvT-13	Significant differences in appearance; unclear boundaries.	VAFormer reduces interference from non-polyp regions; BAFormer optimizes boundary segmentation.	Segmentation performance decreases for polyps that are too small (<5%) or too large (>25%).
		PP-SAM [97]	ViT	Dependence on large amounts of labeled data	The PP-SAM framework enhances SAM’s robustness in polyp segmentation.	Only supports binary classification and single bounding box prompts.
	2023	Polyp-PVT [93]	PVTv2	Differences between features at different levels; feature fusion.	Strong feature extraction capability.	Boundary detection has its limitations.
		PVT- CASCADE [27]	PVTv2 + TransUNet	Limitations of Transformer models.	Multi-stage loss and feature aggregation framework.	Generalization requires more experiments.
		CAFE-Net [94]	PVTv2	The ability to aggregate multi-scale features is limited.	The MFA module maximizes the utilization of previously learned features.	The efficiency of calculations needs improvement.
		DSNet [137]	PVTv2	False positive/negative interference.	Robust performance.	False positives can occur with multiple image noise.
		LACINet [95]	Shunted Transformer-S	Complex backgrounds interfere with pixel-level prediction performance.	The LPM mechanism effectively reduces noise interference and redundant information.	False detections or missed detections may occur under low light or overexposure conditions.
		TransRUPNet [98]	pvt_v2_b2	Real-time polyp segmentation.	Real-time processing speed 47.07 FPS.	Performance significantly declines on out-of-distribution datasets.
	2022	MSRAformer [89]	Swin Transformer	Distinguish between polyp area and boundary.	Enhance the edge details of the segmentation target.	There is still room for improvement in the segmentation of small lesion areas.
		SSFormer [26]	PVTv2	The polyps vary in size and have complex shapes; their borders are unclear.	Strong generalization ability; progressive local decoder.	The findings on previously unseen medical images still require further validation.
		DS-TransUNet [100]	Swin	Ignored the pixel-level intrinsic structural features within each block.	Dual-scale encoding mechanism simultaneously captures coarse-grained and fine-grained features.	Performance is limited when using the same patch size.
Hybrid Architecture	2024	CIFG-Net [111]	PVTv2	The boundaries are unclear.	Improved mDice by 3.9% and mIoU by 4.2% compared to PraNet.	Inference speed and resource consumption not mentioned.
		MVOA-Net [101]	PVTv2-b2	Intra-class inconsistency; inter-class indistinction.	Skilled in multi-target polyp segmentation.	The computational efficiency is not competitive.
		RSAFormer [102]	PVTv2-B4	Boundary similarity.	RSA module can better extract boundary information.	Performs poorly when samples have reflections and shadows.
		Polyper [112]	Swin-T	Fuzzy Boundary.	The segmentation effect for small polyps (image proportion <6%) is good.	Unable to handle false positives or false negatives effectively.
		TransNetR [117]	ResNet50	Inefficient real-time processing speeds.	Processing speed reaches 54.60 FPS.	Detection of tiny polyps still has room for improvement.
		PFD-Net [103]	PVTv2	Failed to effectively utilize local details and global semantic information.	Spatial-frequency joint method enhances the local and global features of feature maps.	When the target and background are extremely similar, segmentation performance is poor.
		HD-Former [104]	ResNet-34	Unable to account for multi-level dependencies between spatial and channel dimensions.	Dual Cross-Attention Transformer (DCAT) module is used for multi-level feature fusion.	Increased computational overhead.
		ASPS [115]	ViT-B +MSCAN-L	Ignoring local details.	Enhanced OOD performance and domain generalization.	There is still room for improvement in the segmentation of small polyps.
		PGCF [138]	PVTv2	Recognition of complex features.	PGCF module extracts multi-scale features.	Inference time is slow, affecting real-time performance.
		FMCA-Net [107]	PVT_v2	Blurred edges; insufficient feature extraction.	The D-BFRM module extracts and enhances polyp features.	The segmentation of small polyps is not ideal.
		SAM2-Unet [116]	Hiera	Design a simple and efficient universal segmentation framework.	Insert an adapter into the encoder to achieve parameter-efficient fine-tuning.	The performance on some datasets is slightly below that of other methods.
		SimTxtSeg [114]	ConvNeXt-Tiny +BERT-BASE	Reducing annotation costs while maintaining segmentation performance.	Text-to-visual prompt converter and text–visual hybrid attention module.	Pseudo-label generation relies on the SAM model and is sensitive to its performance.
	2023	MC-DC [108]	Res2Net-50+Wave-MLP	Design of the feature decoder.	Multi-layer features that integrate MLP and CNN.	The generalization ability needs improvement.
		META-Unet [109]	ResNet34	Low contrast.	The dual-branch structure can simultaneously capture global and local features.	The accuracy of polyp segmentation with ambiguous boundaries is low.
	2022	SwinPA-Net [105]	Swin-B	The size and type of lesions vary too much.	The dense multiplicative connection module is used for multi-scale feature fusion.	Real-time constraints.
		HSNet [106]	PVTv2+ Res2Net50	Ignore the visual details of small polyps.	Interactive attention mechanism; MSP module integrates different scales.	Detection of small polyps still needs improvement.
		PPFormer [113]	CvT+VGG-16	Transformer has insufficient local feature extraction.	PP-guided self-attention mechanism; local-to-global mechanism.	Small polyp segmentation is weak.
		FCBFormer [110]	PVTv2-B3	Only able to predict low-resolution segmentation maps.	FCN and Transformer parallel branches.	Data dependency.
Mamba	2024	VM-UNetV2 [119]	VSS	Limitations of CNN and Transformer.	VSS blocks capture long-range dependencies.	Robustness still needs to be further validated.
		Prompt-Mamba [118]	vision-mamba	Polyps of various shapes and colors.	Image feature extraction in Visual Mamba.	Performance is slightly lower on some datasets.
		Polyp-Mamba [121]	ResNet34+ Mamba	The polyp has blurred margins.	Mamba and ResNet extract global and local features.	Misjudgments may occur in situations with clear boundaries.
		Polyp-Mamba [29]	VSS	Cross-scale dependencies and the consistency of feature representations and semantic embeddings	The SAS module facilitates the interaction of multi-scale semantic information.	Limited validation exists for segmentation of small or complex polyp structures
		H-vmunet [122]	N/A	Defects of the SS2D module.	The H-SS2D module reduces redundant information.	Lack of analysis of model robustness.
Other	2024	SAM 2 [125]	N/A	Depends on extensive dataset labeling.	Strong zero-shot learning capabilities.	The effects vary significantly between different prompts.
		DDPM [124]	U-Net ϵθ	Efficient and accurate polyp segmentation.	Majority voting strategy enhances performance.	Generalization performance needs improvement.
		dHBLSN [126]	BLS(Broad Learning System)	Cost calculation.	BLS does not require multiple layers to learn complex features.	Difficult to remove complex flat polyps.
		HDM [123]	Diffusion U-Net	Domain disparities; low efficiency.	Generation of prior features for polyp segmentation using diffusion models.	Unclear boundaries are difficult to accurately segment.

**Table 3:** Summary of polyp segmentation methods from 2018 to 2021.

Architecture	Year	Methods	Backbone	Problems Solved	Advantages	Limitations
Hybrid Architecture	2021	TransFuse [21]	ResNet-34+ DeiT -S	Efficiency in modeling the global context; low-level details.	Parallel branches; the BiFusion module fuses multi-level features.	Performance on extremely small target datasets has not been discussed in detail.
CNN	2020	PraNet [20]	Res2Net	Polyps exhibit diverse shapes and have indistinct margins.	Parallel partial decoder and reverse attention module.	Detail segmentation remains inadequate in complex boundary situations.
CNN	2019	ResUNet++ [19]	ResUNet	How to accurately segment polyps.	ASPP improves the ability to segment polyps of various shapes and sizes.	Resizing the image may cause some details to be lost.
CNN	2018	Unet++ [18]	UNet	There are semantic differences.	Re-designed skip pathways.	High computational complexity.

**Table 4:** Summary of video polyp segmentation methods.

Architecture	Year	Methods	Backbone	Problems Solved	Advantages	Limitations
CNN	2022	PNS+ [22]	Res2Net50	Lack of large-scale datasets with fine-grained segmentation annotations.	Constructed the SUN-SEG dataset; achieved an inference speed of 170 frames per second.	Insufficient robustness.
CNN	2024	SSTFB [131]	Res2Net-50	Decline in video polyp segmentation performance.	Enhancing representation learning with a spatio-temporal self-attention mechanism.	Some mis-segmentation may occur in complex backgrounds.
Mamba	2024	Vivim [133]	Temporal Mamba Block	Vanilla SSMs cannot preserve non-causal spatial information.	Temporal Mamba Block and ST-Mamba Module.	The model is relatively complex.
Hybrid	2024	MAST [130]	PVTv2-B2	Modeling long-range spatiotemporal relationships is challenging.	The Siamese Transformer and hybrid attention module capture the spatiotemporal relationships between video frames.	Generalization ability needs to be verified.
Hybrid	2024	FlowICBNet [128]	PVT	Predictive discontinuity problems caused by low-quality frames.	RFS and FGW modules select the optimal historical reference frame.	The IFU exhibits an over-correction phenomenon.
Hybrid	2024	SALI [132]	PVTv2	Low-quality frames limit segmentation accuracy.	The SAM module alleviates spatial variations; the LIM module reconstructs polyp features.	Segmentation remains inaccurate for frames with drastic changes.
Hybrid	2024	Diff-VPS [129]	N/A	Highly camouflaged polyps and redundant temporal clues.	Multi-task diffusion model; the TRM module captures dynamic video features.	There is still room for improvement in detecting highly concealed polyps.

**Table 6:** Definitions of TP, FP, TN, and FN.

Indicator	Definition	Meaning
True Positive (TP)	Correctly predicted as positive class.	It is a polyp and has been correctly predicted.
False Positive (FP)	Incorrectly predicted as positive class.	Falsely predicted as a polyp, but not actually a polyp.
True Negative (TN)	Correctly predicted as negative class.	Not a polyp and correctly predicted.
False Negative (FN)	Incorrectly predicted as negative class.	It is a polyp but was incorrectly predicted not to be a polyp.

**Table 7:** Comparative study of performance evaluation of various models for polyp segmentation. The top 2 methods are marked in Green and Red, respectively.

Method	CVC-ClinicDB		Kvasir-SEG		CVC-ColonDB		ETIS-Larib		EndoScene	
	mDice	mIoU	mDice	mIoU	mDice	mIoU	mDice	mIoU	mDice	mIoU
MEGANet [63]	0.938	0.894	0.913	0.863	0.793	0.714	0.739	0.665	0.899	0.834
Polyper [112]	0.945	0.899	0.948	0.904	0.837	0.746	0.865	0.785	0.924	0.867
Polyp-Mamba [29]	0.949	0.907	0.940	0.881	0.829	0.743	0.825	0.747	0.921	0.875
ISCNet [74]	0.961	0.920	0.939	0.898	0.828	0.741	0.804	0.716	0.871	0.778
CTNet [28]	0.936	0.887	0.917	0.863	0.813	0.734	0.810	0.734	0.908	0.844
PraNet [20]	0.899	0.849	0.898	0.840	0.709	0.640	0.628	0.567	0.871	0.797
CFA-Net [66]	0.933	0.883	0.915	0.861	0.743	0.665	0.732	0.655	0.893	0.827
DLCA_CMAM [69]	0.944	0.900	0.929	0.882	0.818	0.745	0.839	0.766	0.903	0.837
Polyp-PVT [93]	0.937	0.889	0.917	0.864	0.808	0.727	0.787	0.706	0.900	0.833
CAFE-Net [94]	0.943	0.899	0.933	0.889	0.820	0.740	0.822	0.738	0.901	0.834
UNet [15]	0.823	0.755	0.818	0.746	0.512	0.444	0.398	0.335	0.710	0.627
TGANet [136]	0.946	0.899	0.898	0.833	0.755	0.824	0.636	0.782	0.885	0.899
HST-MRF [99]	0.935	0.884	0.928	0.885	0.831	0.776	0.773	0.719	0.901	0.846
DS-TransUNet [100]	0.936	0.887	0.935	0.889	0.798	0.722	0.761	0.687	0.911	0.846
TransFuse [21]	0.942	0.897	0.920	0.870	0.781	0.706	0.737	0.663	0.894	0.826
MVOA-Net [101]	0.947	0.902	0.935	0.891	0.824	0.745	0.820	0.743	0.904	0.838
SwinPA-Net [105]	0.941	0.894	0.925	0.876	0.807	0.726	0.762	0.684	0.893	0.823
HSNet [106]	0.948	0.905	0.926	0.877	0.810	0.735	0.808	0.734	0.903	0.839
SAM 2 [125]	0.930	0.870	0.939	0.885	0.934	0.877	0.941	0.890	-	-
PFD-Net [103]	0.939	0.893	0.928	0.880	0.816	0.737	0.826	0.746	-	-
CoInNet [73]	0.930	0.887	0.926	0.872	0.797	0.729	0.759	0.690	-	-
AMNet [78]	0.936	0.888	0.912	0.865	0.762	0.690	0.756	0.679	-	-
DEC-Seg [80]	0.859	0.804	0.893	0.830	0.721	0.640	0.634	0.564	-	-
WS-DefSegNet [81]	0.807	0.746	0.768	0.709	0.667	0.588	0.596	0.517	-	-
NPD-Net [82]	0.925	0.876	0.905	0.850	0.764	0.682	0.737	0.659	-	-
MISNet [64]	0.918	0.869	0.903	0.846	0.762	0.690	0.764	0.686	-	-
UHA-Net [70]	0.927	0.881	0.908	0.857	0.769	0.695	0.746	0.670	-	-
SEPNet [71]	0.937	0.887	0.922	0.869	0.819	0.740	0.795	0.718	-	-
FEGNet [72]	0.943	0.901	0.923	0.874	0.767	0.686	0.719	0.645	-	-
MLFF-Net [90]	0.943	0.897	0.919	0.866	0.820	0.742	0.784	0.707	-	-
PSTNet [91]	0.945	0.901	0.935	0.895	0.827	0.748	0.800	0.726	-	-
PFENet [92]	0.940	0.897	0.931	0.886	0.821	0.745	0.809	0.735	-	-
FMCA-Net [107]	0.944	0.898	0.919	0.866	0.804	0.723	0.841	0.765	-	-
FCBFormer [110]	0.947	0.902	0.939	0.890	0.783	0.706	0.796	0.715	-	-
CIFG-Net [111]	0.938	0.891	0.925	0.876	0.815	0.733	0.806	0.726	-	-
SAM2-UNet [116]	0.907	0.856	0.928	0.879	0.808	0.730	0.796	0.723	-	-
Prompt-Mamba [118]	0.888	0.814	0.886	0.808	0.820	0.712	0.771	0.663	-	-
VM-UNetV2 [119]	0.944	0.893	0.913	0.842	0.758	0.610	0.839	0.723	-	-
Polyp-Mamba [121]	0.941	0.896	0.919	0.867	0.791	0.713	0.756	0.668	-	-
RPANet [88]	0.800	0.719	0.858	0.782	-	-	0.632	0.552	-	-
TransNetR [117]	0.766	0.691	0.871	0.802	-	-	-	-	-	-
DDPM [124]	0.967	0.937	0.934	0.886	-	-	-	-	-	-
TransRUPNet [98]	0.854	0.777	0.901	0.845	-	-	-	-	-	-
Polyp-LVT [3]	0.935	0.882	0.909	0.851	-	-	-	-	0.904	0.835

**Table 8:** Comparative study of various models in terms of real-time performance.

Models	Params (M)	Flops (G)	FPS (Frames/s)
MISNet [64]	33.63	45.94	30.68
CFA-Net [66]	25.24	55.36	23.50
SEPNet [71]	25.96	12.52	62.00
TGANet [136]	42.30	19.84	85.00
MSRF-Net [76]	18.38	20.26	14.38
I-RA [77]	35.85	15.65	2.06
PraNet [20]	30.50	13.08	37.31
LACINet [95]	22.36	11.74	28.41
MVOA-Net [101]	27.86	29.73	30.78
TransNetR [117]	27.27	10.58	54.60
Polyp-Mamba [121]	49.50	27.90	23.80
ERDUnet [23]	10.21	10.30	27.03
PolypSeg+ [127]	2.54	7.23	31.00
FlowICBNet [128]	98.40	N/A	29.00
SSTFB [131]	33.40	N/A	126.00
UNet [15]	34.52	N/A	55.10
WDFF-Net [24]	17.46	N/A	83.82
META-Unet [109]	27.32	N/A	75.00
Polyp-PVT [93]	125.60	N/A	66.00
MC-DC [108]	65.93	41.13	N/A
FTMF-Net [79]	44.77	8.85	N/A
Polyp-LVT [3]	25.11	13.21	N/A
NPD-Net [82]	29.22	14.51	N/A
CTNet [28]	44.19	15.20	N/A
FMCA-Net [107]	28.61	14.36	N/A
PFD-Net [103]	28.89	16.81	N/A
ISCNet [74]	23.34	15.57	N/A
CAFE-Net [94]	35.53	16.12	N/A
DS-TransUNet [100]	287.75	51.09	N/A
MAST [130]	25.69	21.02	N/A
Prompt-Mamba [118]	102.00	N/A	N/A
HSNet [106]	29.23	N/A	N/A
MEGANet [63]	44.19	N/A	N/A
TransFuse [21]	26.30	N/A	N/A
HST-MRF [99]	174.73	N/A	N/A
TransRUPet [98]	N/A	N/A	47.07

Note: N/A indicates data not available.

## Data Availability

The datasets referenced and analyzed in this review are publicly available from their original sources: CVC-ColonDB: https://www.kaggle.com/datasets/longvil/cvc-colondb (accessed on 1 December 2024.); ETIS-Larib: https://drive.google.com/drive/folders/10QXjxBJqCf7PAXqbDvoceWmZ-qF07tFi (accessed on 1 December 2024.); CVC-ClinicDB: https://polyp.grand-challenge.org/CVCClinicDB/ (accessed on 1 December 2024.); CVC-EndoSceneStill: https://drive.google.com/file/d/1MuO2SbGgOL_jdBu3ffSf92feBtj8pbnw/view (accessed on 1 December 2024.); Kvasir-SEG: https://datasets.simula.no/kvasir-seg/ (accessed on 1 December 2024.); PICCOLO: https://www.biobancovasco.bioef.eus/en/Sample-and-data-catalog/Databases/PD178-PICCOLO-EN.html (accessed on 1 December 2024.); Hyper-Kvasir: https://datasets.simula.no/hyper-kvasir/ (accessed on 1 December 2024.); BKAI-IGH: https://www.kaggle.com/competitions/bkai-igh-neopolyp/data (accessed on 1 December 2024.); PolypGen: https://www.synapse.org/Synapse:syn26376615/files/ (accessed on 1 December 2024.); LDPolypVideo: https://github.com/dashishi/LDPolypVideo-Benchmark (accessed on 1 December 2024.); Kvasir-Capsule: https://osf.io/dv2ag/ (accessed on 1 December 2024.); SUN-SEG: https://github.com/GewelsJI/VPS/blob/main/docs/DATA_PREPARATION.md (accessed on 1 December 2024.).

## Abbreviations

The following abbreviations are used in this manuscript:

AP	Average Precision
BSCA	Bit-Slice Context Attention
BWM	Balanced Weight Module
CMAM	Convolutional Multi-Scale Attention Module
CNN	Convolutional Neural Network
CRC	Colorectal Cancer
DCL	Domain Contrastive Learning
DCT	Discrete Cosine Transform
DDPM	Denoising Diffusion Probabilistic Model
DLCA	Dataset-Level Color Augmentation
DPC	Dual-Path Complementary
DSC	Dice Similarity Coefficient
FCNs	Fully Convolutional Networks
FGW	Flow-Guided Warping
FoBS	Focus on Boundary Segmentation
FPS	Frames Per Second
FT	Fourier Transform
GFlops	Giga Floating Point Operations Per Second
IFU	Iterative Feedback Unit
IoU	Intersection over Union
MACE	Multi-Path Attention Encoder
MAST	Mixture-Attention Siamese Transformer
MCAD	Multi-Path Attention Decoder
mDice	mean Dice coefficient
mIoU	mean Intersection over Union
MLP	Multi-Layer Perceptron
PAM	Parallel Attention Module
PVTv2	Pyramid Vision Transformer v2
RFS	Reference Frame Selection
RSA	Region Self-Attention
SAM	Segment Anything Model
SAM2	Segment Anything Model 2
SSBU	Segmentation-Squeeze-Bottleneck Union
SSFM	Selective Shared Fusion Module
SSM	State Space Model
TRM	Temporal Reasoning Module
ViT	Vision Transformer
VSS	Visual State Space
WoS	Web of Science
